# SARS-CoV-2 host cell entry: an in silico investigation of potential inhibitory roles of terpenoids

**DOI:** 10.1186/s43141-021-00209-z

**Published:** 2021-08-05

**Authors:** Gideon A. Gyebi, Oludare M. Ogunyemi, Ibrahim M. Ibrahim, Olalekan B. Ogunro, Adegbenro P. Adegunloye, Saheed O. Afolabi

**Affiliations:** 1 Department of Biochemistry, Faculty of Sciences and Technology, Bingham University, P.M.B 005, Karu, Nasarawa State Nigeria; 2 Human Nutraceuticals and Bioinformatics Research Unit, Department of Biochemistry, Salem University, Lokoja, Nigeria; 3 Faculty of Sciences, Department of Biophysics Cairo University, Giza, Egypt; 4 Department of Biological Sciences, KolaDaisi University, Ibadan, Nigeria; 5 Department of Biochemistry, Faculty of Life Sciences, University of Ilorin, Ilorin, Nigeria; 6 Department of Pharmacology and Therapeutics, Faculty of Basic Medical Sciences, University of Ilorin, Ilorin, Nigeria

**Keywords:** SARS-CoV-2, ACE2, TMPRSS2, Spike protein, Terpenoids, Abietane diterpenes: Molecular docking

## Abstract

**Background:**

Targeting viral cell entry proteins is an emerging therapeutic strategy for inhibiting the first stage of SARS-CoV-2 infection. In this study, 106 bioactive terpenoids from African medicinal plants were screened through molecular docking analysis against *human* angiotensin-converting enzyme 2 (*h*ACE2), *human* transmembrane protease serine 2 (TMPRSS2), and the spike (S) proteins of SARS-CoV-2, SARS-CoV, and MERS-CoV. In silico absorption-distribution-metabolism-excretion-toxicity (ADMET) and drug-likeness prediction, molecular dynamics (MD) simulation, binding free energy calculations, and clustering analysis of MD simulation trajectories were performed on the top docked terpenoids to respective protein targets.

**Results:**

The results revealed eight terpenoids with high binding tendencies to the catalytic residues of different targets. Two pentacyclic terpenoids (24-methylene cycloartenol and isoiguesteri) interacted with the *h*ACE2 binding hotspots for the SARS-CoV-2 spike protein, while the abietane diterpenes were found accommodated within the S1-specificity pocket, interacting strongly with the active site residues TMPRSS2. 3-benzoylhosloppone and cucurbitacin interacted with the RBD and S2 subunit of SARS-CoV-2 spike protein respectively. These interactions were preserved in a simulated dynamic environment, thereby, demonstrating high structural stability. The MM-GBSA binding free energy calculations corroborated the docking interactions. The top docked terpenoids showed favorable drug-likeness and ADMET properties over a wide range of molecular descriptors.

**Conclusion:**

The identified terpenoids from this study provides core structure that can be exploited for further lead optimization to design drugs against SARS-CoV-2 cell-mediated entry proteins. They are therefore recommended for further in vitro and in vivo studies towards developing entry inhibitors against the ongoing COVID-19 pandemic.

**Supplementary Information:**

The online version contains supplementary material available at 10.1186/s43141-021-00209-z.

## Background

The coronavirus disease-19 (COVID-19) caused by severe acute respiratory syndrome coronavirus 2 (SARS-CoV-2) was declared a public health emergency by the World Health Organization (WHO) [8, 55, 66, 67]. The death toll from this virus has by far surpassed that of 2003 severe acute respiratory syndrome-coronavirus (SARS-CoV) and the 2012 Middle East respiratory syndrome coronavirus (MERS-CoV) outbreaks combined [21, 53]. The SARS-CoV-2 earlier known as 2019 novel coronavirus (2019-nCoV) is evolutionarily related (80% identity) to SARS-CoV [9]. It causes multiple organ failures, which may present as fever, cough, shortness of breath, dyspnea, pneumonia, severe acute respiratory syndrome, kidney failure, and even death [31, 68]. Bioinformatics has proven a notable tool in understanding the virulence and interaction of the SARS-CoV-2 to different receptors [32, 56, 57]. Cell entry of coronaviruses depends on a fine interplay between the viral membrane spike (S) proteins and the host cell membrane proteins more importantly are the angiotensin-converting enzyme 2 (ACE2) and serine protease transmembrane protease serine 2 (TMPRSS2) [7]. The S-protein comprises two subunits; S1 as the receptor-binding domain (RBD) while S2 subunit is for the fusion of viral membrane and host cellular membrane. The SARS-CoV-2 relies on the host ACE2 for entry and the TMPRSS2 for S-protein priming. Upon binding of the S-protein to host receptor through the receptor-binding domain (RBD) in the S1 subunit, the S2 subunit mediates fusion of the viral envelope with the host membranes [12]. Although the overall sequence similarity between S-protein of SARS-COV-2 and SARS-CoV is approximately ~ 76%, affinity between S-RBD of SARS-COV-2 and ACE2 is found to be approximately four fold higher when compared with SARS-CoV RBD [12, 64]. This molecular interaction is responsible for regulating both the cross-species and higher human-to-human transmissions of SARS-CoV-2 [63, 74]. Therefore, these protein effectors of viral attachment, membrane fusion, and cell entry are known as emerging targets for development of entry inhibitors, antibodies, and vaccines [74].

The use of phytomedicines as alternatives to combat viral diseases and other infections forms an integral component of African cultural practices, and hence a prominent feature in Africa [3, 5, 18, 37, 41, 61]. Terpenoids are a well-known class of phytochemicals of tremendous pharmaceutical value over time because of their relevant broad-spectrum utility in medicine [17, 23, 40]. Screening a database of phytochemicals from indigenous African medicinal plants may help identify terpenoids with therapeutic potentials against the COVID-19 pandemic. Therefore, this study explores computational screening of terpenoids from indigenous African medicinal plants as potential inhibitors of the emerging proteins responsible for coronavirus cell entry and subsequent infection.

## Methods

### Protein preparation

The crystal structures of proteins for the docking studies were retrieved from the Protein Databank (http://www.rcsb.org) with their various PDB identification codes [1R42: angiotensin-converting enzyme 2 (ACE2) [58]; 2OQ5: type II transmembrane serine proteinases **(**TMPRSS2) [26]; 6vw1: 2019-nCoV chimeric receptor-binding domain complexed with its receptor human ACE2 (ACE2-RBD) [49] and coronaviruses spike protein (6VSB: SARS-CoV-2) [67]; (5X5B: SARS-CoV) [72] and (5x5c: MERS-CoV) [72]. All the crystal structures were prepared by removing existing ligands and water molecules, missing hydrogen atoms were added while the Kollamn charge were added as the partial atomic charge using MGL-AutoDockTools (ADT, v1.5.6) [36]. The well-ordered scheme was repeated for each protein and thereafter saved into dockable pdbqt format for molecular docking.

### Ligand preparation

One hundred and six bioactive terpenoids from African medicinal plants were collected based on literature search. Structure Data Format (SDF) of the reference inhibitors (S1: MLN-4760; S2: camostat and S3: nelfinavir mesylates) and 106 bioactive terpenoids derived from African plants were retrieved from the PubChem database (www.pubchem.ncbi.nlm.nih.gov) and converted to mol2 chemical format using Open babel [39]. Other compounds that were not available on the database were drawn with Chemdraw version 19 and converted to mol2 chemical format. Polar hydrogen charges of the gasteiger-type were assigned and the nonpolar hydrogen molecules were merged. The ligand molecules were further converted to the dockable pdbqt format using MGL-AutoDockTools (ADT, v1.5.6) [36].

### Molecular docking

Molecular docking was performed to evaluate the binding affinity and to provide initial coordinates and topology parameters for the MD simulations. The screening of human enzymes and active regions of the coronaviruses spike protein and determination of binding affinities were carried out using AutoDock Vina [59]. The binding scores from vina analysis were further validated by BINDSURF [48]. Docking of bioactive terpenoids and reference compounds against human ACE2, human TMPRSS2, and SARS-CoV-2 spike protein was performed by AutoDock Vina to locate alternate binding sites enclosing the whole macromolecules. Default settings of Vina wase used, as the scoring matrix in this program is stochastic, and each run uses a random seed position except for the grid box which was adjusted with extended grid size (60 Å × 60 Å × 60 Å) to reveal all the possible interaction sites. The molecular docking was executed using a flexible docking protocol; all bonds contained in ligand were allowed to rotate freely, making the receptor rigid. Once the molecular docking experiments were completed and 10 configurations for each protein-ligand complex were generated for all the terpenoids, text files of scoring results were also produced for the purpose of manual comparative analysis. The top docked terpenoids were uploaded into the respective columns of BINDSURF webserver to validate and calculate the energetic interactions. The molecular interactions between proteins and selected compounds with higher binding affinity to the proteins were viewed with Discovery Studio Visualizer version 16.

### Molecular dynamics simulation

Molecular dynamics simulations were carried out on the top ranked terpenoid to respective protein targets (SARS-CoV-2 spike (S) protein, human angiotensin-converting enzyme 2 (ACE2), and transmembrane protease serine 2 (TMPRSS2)). The complexes were prepared and solvated, in TIP3P water model and neutralized by adding NaCl ions and its concentration was set to 0.154 M using CHARMM-GUI webserver prior to running MD simulation using Nanoscale Molecular Dynamics (NAMD V 2.13) [6, 27, 44]. The ligands (terpenoids) were parameterization on the SwissParam webserver. The TIP3P water model was used to resemble the added water box, with 10 Å padding, for the periodic boundary condition to be applied [34]. Nose-Hoover Langevin piston was used to control the pressure at 1.01325 bar. In contrast, Langevin dynamics controlled the system’s temperature at the physiological value (310 K, 7.0, and 0.154 M NaCl, respectively). The time step was set at its default two fs with SHAKE approximation. Visualizing molecular dynamics (VMD 1.9.3) software was used to prepare the input files and analyze the output trajectories [22]. Minimization step for the complexes was initiated for 10,000 steps using a conjugate gradient algorithm in constant number of atoms, constant volume, and constant temperature ensemble (NVT) using CHARMM 36 force field. Afterwards, equilibration of each system for one nanosecond was started in constant number of atoms, constant pressure, and constant temperature ensemble (NPT). Finally, a production run for 100 ns for each system was initialized in NVT ensemble. Periodic Boundary Conditions (PBC) was applied to the simulation. Trajectories were extracted each 0.1 ns and time step was set to 2 femto second. The analysis of the dynamics was performed by utilizing VMD scripts to calculate root mean square deviation (RMSD), root mean square fluctuation (RMSF), surface accessible surface area (SASA), radius of gyration (RoG), and hydrogen bonds (H-bonds) [22]. All the analyses were performed after removing the PBC using the pbctools package in VMD using this command pbc unwrap-sel “selection” where selection is replaced by the appropriate name.

### Clustering of molecular dynamic trajectory

Afterwards, TTClust V 4.9.0 [60] was used to cluster the whole trajectory (1000 frame) using the elbow method to calculate the optimum number of clusters. For each representative frame produced, Protein Ligand Interaction Profiler (PLIP) [47] was used to know the types and number of interactions between the protein and the ligand.

### MM/GBSA calculation and MM/GBSA free energy decomposition analysis

To calculate the binding free energies of the top docked terpenoids to each of the protein target, molecular mechanics–generalized born surface area (MM-GBSA) was calculated using the version implemented in AmberTools 20 for all frames in the trajectory [35, 54]. Saltcon variable was set to 0.154 M and igb, which determines the generalized born method to use, was set to the default value of five. After the decomposition process, the energy contribution could be distributed to each residue of receptor and the binding interaction of each ligand-residue pair consists of three energy terms: van der Waals contribution (Δ*E*_vdw_), electrostatic contribution (Δ*E*_ele_), and the desolvation term (Δ*G*_desolvation_) which included the polar (Δ*G*_GB_), the non-polar (Δ*G*_SA_), and total free energy (∆*G*_total_) term. Fifty frames separated by equal intervals of 20 frames were used to generate the binding free energies and were also used for the free energy decomposition analysis.

### Drug-likeness and ADMET studies

The top terpenoids that demonstrated highest binding affinity for ACE2, TMPRSS2, and active regions of SARS-CoV-2 spike protein were subjected to several drug-likeness predictive descriptors which orally bio-active drug should comply [30, 38]. The predicted absorption, distribution, metabolism, excretion, and toxicity (ADMET) studies were analyzed using the ADMET webserver [10]. The SDF file and SMILES of the compounds were downloaded from PubChem database to calculate ADMET properties using default parameters.

## Results

### Molecular docking

**Fig. 1:**
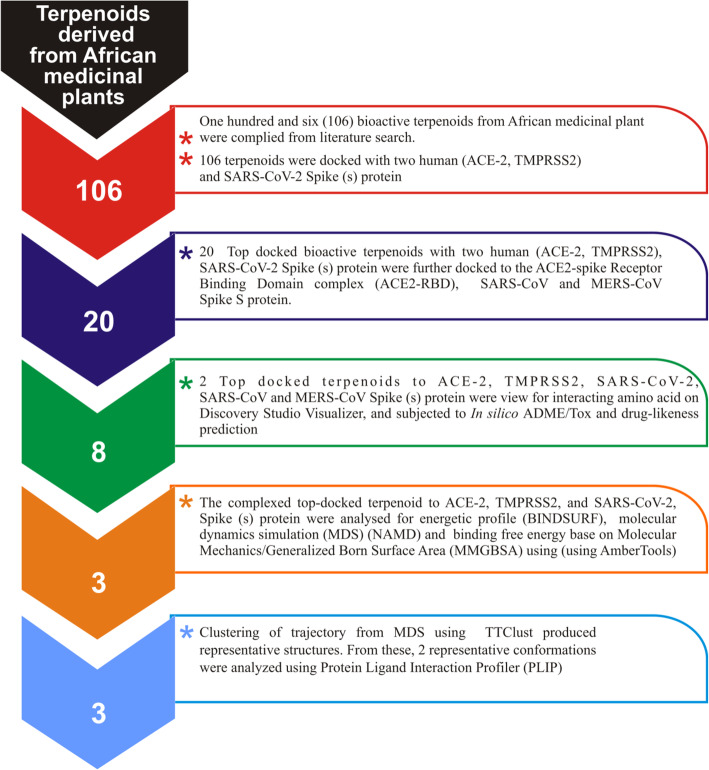
Flow chart showing the stepwise screening of African derived terpenoids for potential inhibitors of membrane-mediated SARS-CoV-2 cell entry

Figure 1 provides a flow chart showing the stepwise screening of African derived terpenoids for potential inhibitors of SARS-CoV-2 cell entry proteins.

**Fig. 2:**
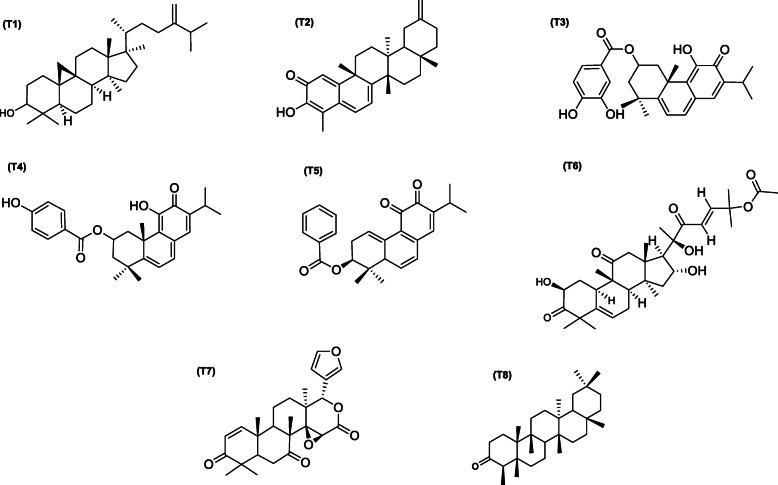
Chemical structure of terpenoid with remarkable binding energy to human ACE2, TMPRSS2, and SARS-coronaviruses S protein (**T1**) 24-methylene cycloartenol; (**T2**) Isoiguesterin; (**T3**) 11-hydroxy-2-(3,4-dihydroxybenzoyloxy)abieta-5,7,9(11),13-tetraene-12-one; **(T4)** 11-Hydroxy-2-(4-hydroxybenzoyloxy)-abieta-5,7,9(11),13-tetraene-12-one; (**T5**) 3-benzoylhosloppone; (**T6**) Cucurbitacin B; (**T7**) 7-deacetoxy-7-oxogedunin and (**T8**) 3-Friedelanone

The result from the docking analysis of the reference inhibitors and bioactive terpenoids with the human ACE2, TMPRSS2, and SARS-CoV-2 spike protein is shown in Table S1 (supplementary material). The top 20 terpenoids with the highest binding affinity for the ACE2 were further analyzed for binding interactions with SARS-CoV-2 chimeric receptor-binding domain complexed with its human receptor ACE2 (ACE2-RBD) and the S protein of SARS-CoV and MERS-CoV (Table S3, supplementary material) (Fig. 2).

**Fig. 3:**
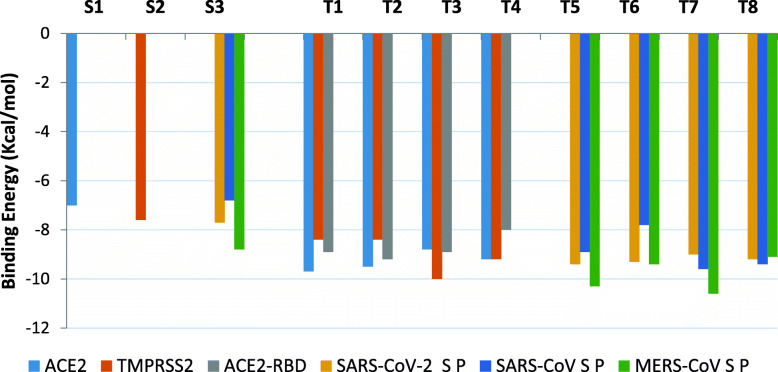
AutoDock binding energies (Kcal/mol) of reference inhibitors and top bioactive terpenoids with *human* angiotensin-converting enzyme 2 (ACE2), transmembrane protease serine 2 (TMPRSS2), ACE2-SARS-CoV-2 spike receptor binding domain complex (ACE2-RBD), and (*S P) spike protein of coronaviruses. **S1** MLN-4760. **S2** Camostat. **S3** Nelfinavir mesylates. **T1** 24-methylene cycloartenol. **T2** Isoiguesterin. **T3** 11-hydroxy-2-(3,4-dihydroxybenzoyloxy)abieta-5,7,9(11),13-tetraene-12-one. **T4** 11-hydroxy-2-(4-hydroxybenzoyloxy)-abieta-5,7,9(11),13-tetraene-12-one. **T5** 3-benzoylhosloppone. **T6** Cucurbitacin B. **T7** 7-deacetoxy-7-oxogedunin. **T8** 3-Friedelanone

The docking analysis revealed that the reference inhibitor (MLN-4760) to the human ACE2 protein had binding energy of − 7.7 Kcal/mol, respectively, while camostat an inhibitor of TMPRSS2 had a binding energy of − 7.6 Kcal/mol as represented in Fig. 3. It was further observed that the topmost docked terpenoids to the ACE2 had higher binding affinity for the S protein of SARS-CoV and MERS-CoV than SARS-CoV-2. More than 10 terpenoids had higher binding affinity than the 3 inhibitors used in this study (Table S1: supplementary material). The top 20 docked compounds to SARS-CoV-2 S-proteins had higher binding affinity than nelfinavir mesylates (Table S3: Supplementary material).

From the binding scores generated by the interacting terpenoids with the ACE2 and TMPRSS2 proteins, the top 2 docked terpenoids with the highest binding affinity are 24-methylene cycloarteno and isoiguesterin with corresponding binding energy of − 9.7, and − 9.5 Kcal/mol, respectively. The best two docked terpenoids to SARS-CoV-2 S protein are 3-benzoylhosloppone and cucurbitacin with binding energies of − 9.4 and − 9.3 Kcal/mol respectively. 3-benzoylhosloppone had the highest binding affinity for SARS-CoV-2 S protein and the second top binding affinity to MERS-CoV S protein (Fig. 3).

#### Interaction of selected terpenoids with amino acids of target proteins

**Fig. 4:**
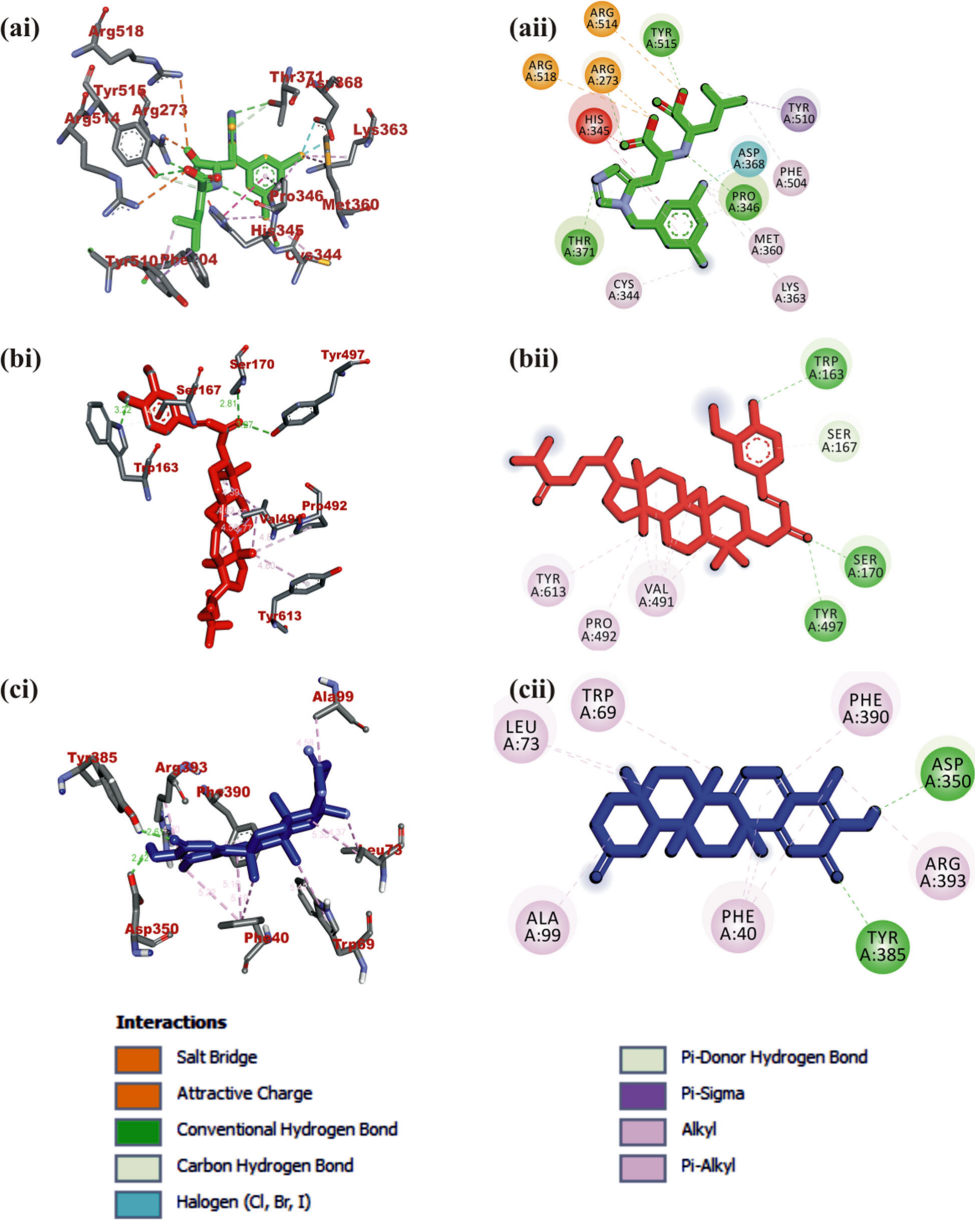
Visualization of interacting amino acid residues of *human* ACE2 with ligands in 3D (**i**) and 2D (**ii**) representation. Ligands in stick representation are presented in different colors. **a** Green: S1 (MLN-4760). **b** Read: 24-methylene cycloartenol. **c** Blue: isoiguesterin. Types of interactions are represented by green-dotted lines: H-bond interactions, light purple-dotted line: hydrophobic interactions (Pi-Alkyl, Alkyl, and pi-stacking) purple-dotted line: Pi-Pi T-shaped, yellow-dotted lines: Pi-sulphur interactions, pi-stacking interactions. Three-letter amino acids are in red color

**Fig. 5:**
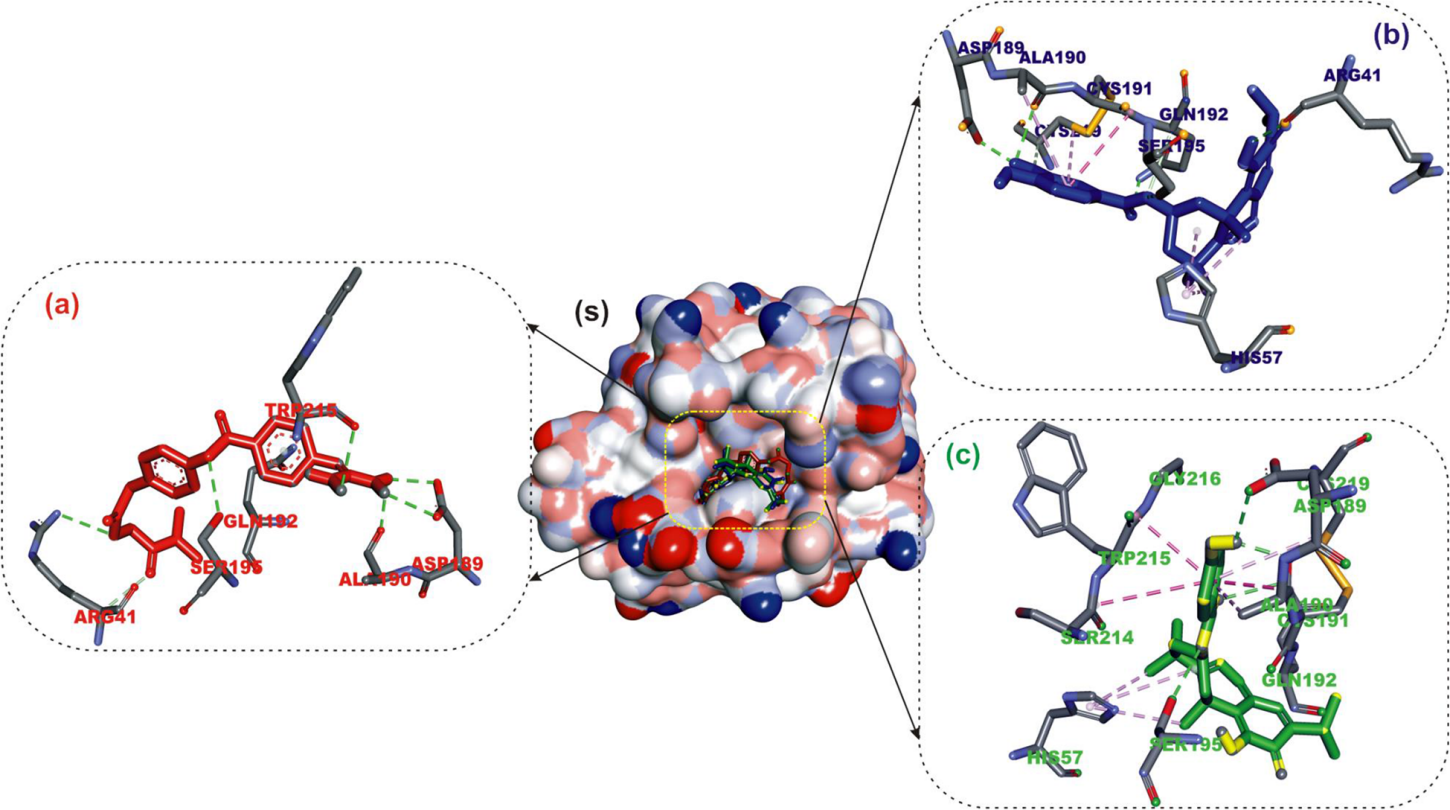
Amino acid interactions of terpenoids in substrate binding cavity of human TMPRSS2. **S** Surface representation. Ligands in sticks representation are represented by colors. **a** Red: camostat. **b** Blue: 11-hydroxy-2-(3,4-dihydroxybenzoyloxy)abieta-5,7,9(11),13-tetraene-12-one. **c** Green: 11-hydroxy-2-(4-hydroxybenzoyloxy)-abieta-5,7,9(11),13-tetraene-12-one. Types of interactions are represented by green-dotted lines: H-bond interactions, light purple-dotted line: hydrophobic interactions (Pi-Alkyl, Alkyl, and pi-stacking) purple-dotted line: Pi-Pi T-shaped, yellow-dotted lines: Pi-sulphur interactions, pi-stacking interactions

**Fig. 6:**
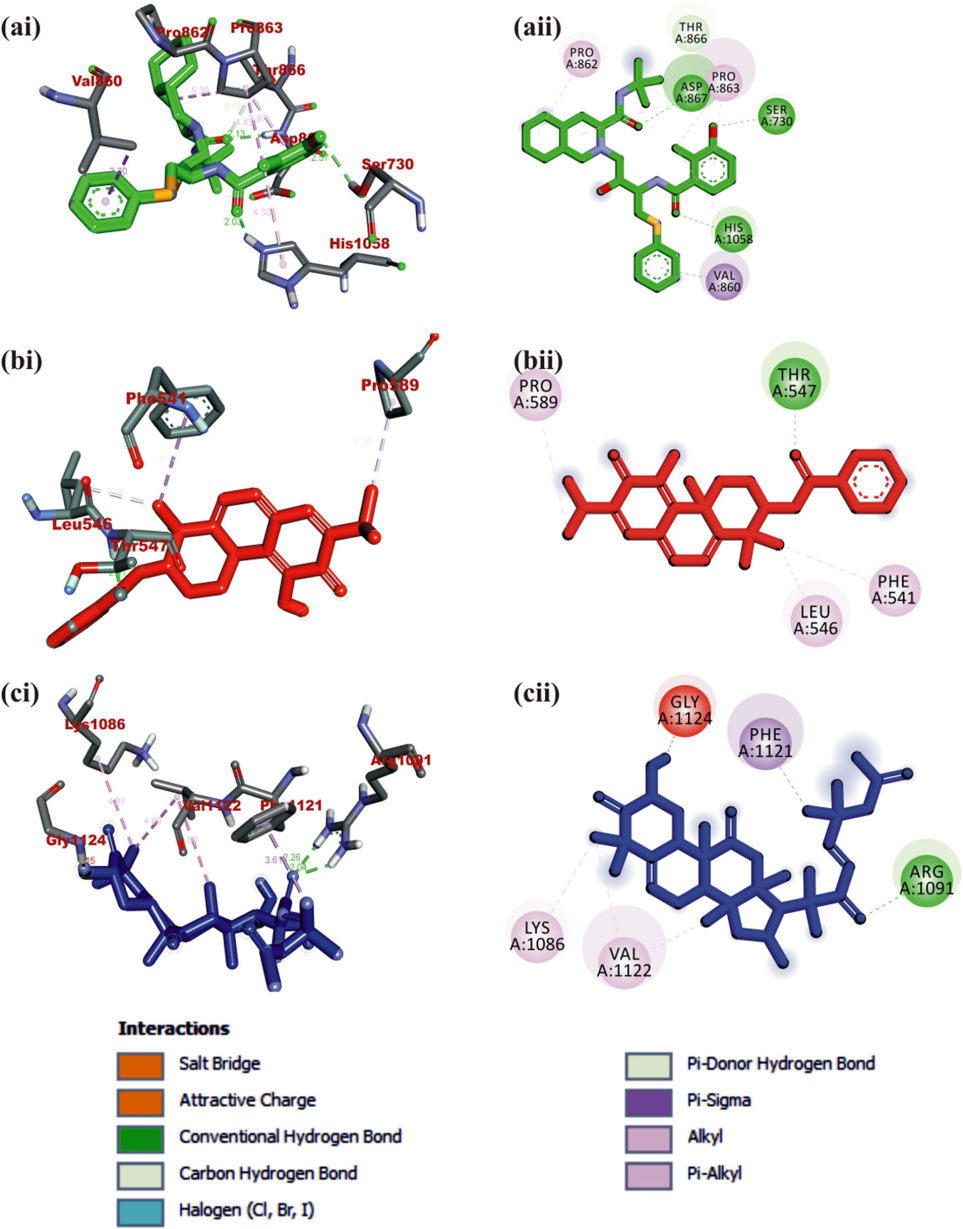
Visualization of interacting amino acid residues of SARS-CoV-2 spike protein with ligands in 3D (i) and 2D (ii) representation. Ligands in stick representation are presented in different colors. **a** Green: nelfinavir mesylates (S3). **b** Red: 3-benzoylhosloppone. **c** Blue: cucurbitacin B. Types of interactions are represented by green-dotted lines: H-bond interactions, light purple-dotted line: hydrophobic interactions (Pi-Alkyl, Alkyl, and pi-stacking) purple-dotted line: Pi-Pi T-shaped, yellow-dotted lines: Pi-sulphur interactions, pi-stacking interactions

**Fig. 7:**
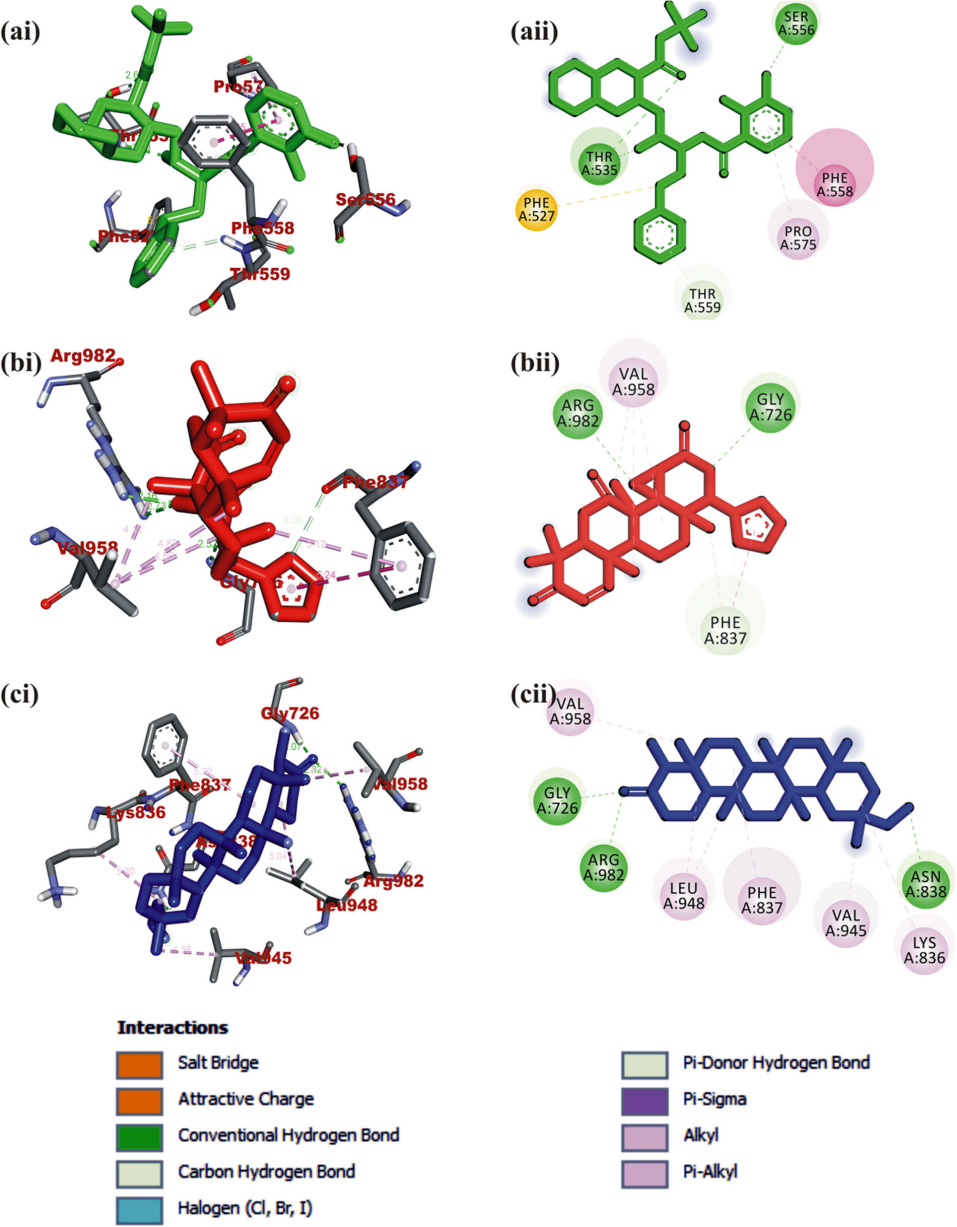
Visualization of interacting amino acid residues of SARS-CoV spike protein with ligands in 3D (i) and 2D (ii) representation. Ligands in stick representation are presented in different colors. **a** *Green*: nelfinavir mesylates (S3) **b** Red: 7-deacetoxy-7-oxogedunin. **c** Blue: 3-friedelanone.. Types of interactions are represented by green-dotted lines: H-bond interactions, light purple-dotted line: hydrophobic interactions (Pi-Alkyl, Alkyl, and pi-stacking) purple-dotted line: Pi-Pi T-shaped, yellow-dotted lines: Pi-sulphur interactions, pi-stacking interactions

**Fig. 8:**
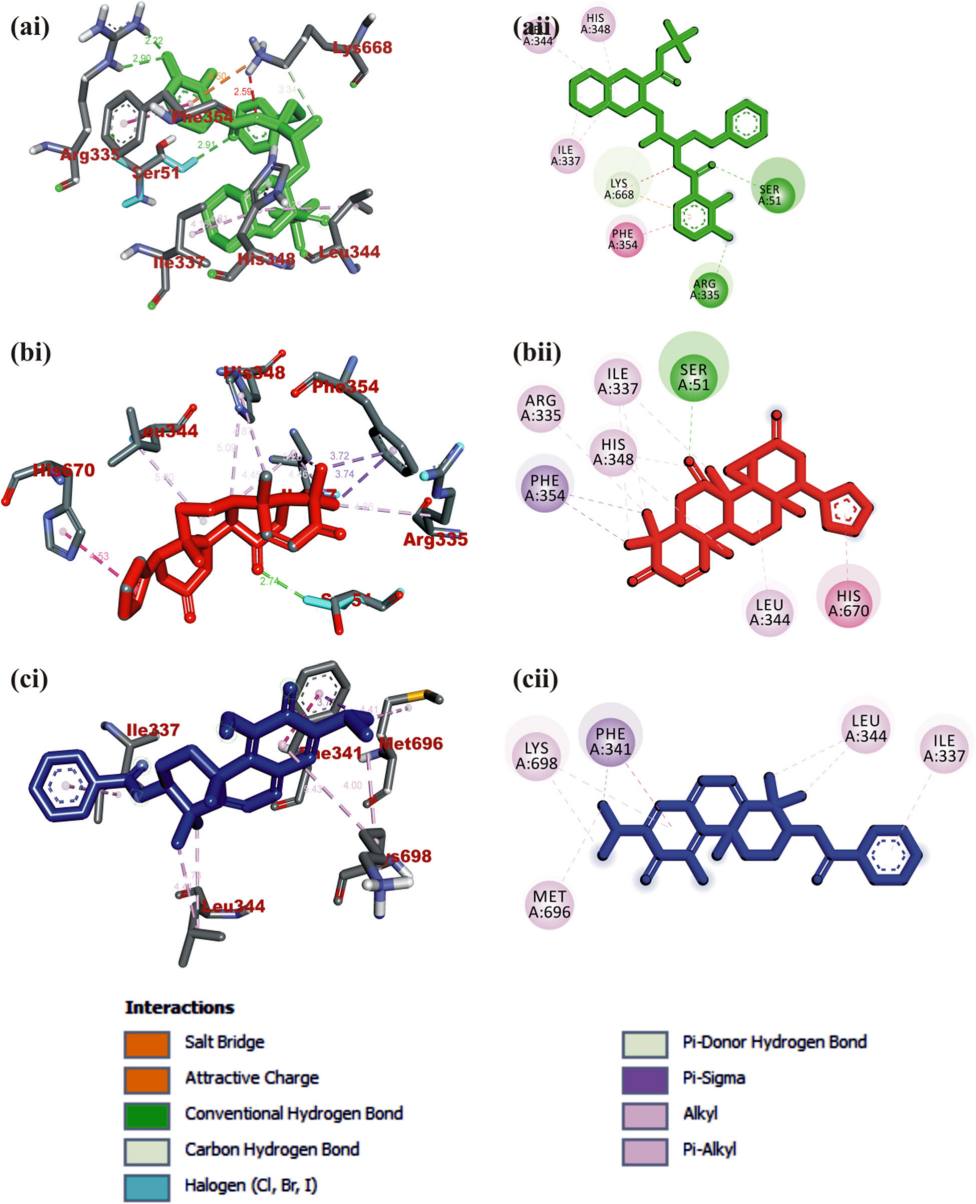
Visualization of interacting amino acid residues of MERS-CoV spike protein with ligands in 3D (i) and 2D (ii) representation. Ligands in stick representation are presented in different colors. **a** Green: nelfinavir mesylates (S3). **b** Red: 7-deacetoxy-7-oxogedunin. **c** Blue: 3-benzoylhosloppone. Types of interactions are represented by green-dotted lines: H-bond interactions, light purple-dotted line: hydrophobic interactions (Pi-Alkyl, Alkyl, and pi-stacking) purple-dotted line: Pi-Pi T-shaped, yellow-dotted lines: Pi-sulphur interactions, pi-stacking interaction

The amino acid interactions of the *human* target proteins (ACE2 and TMPRSS2) with reference inhibitors and plant derived terpenoids that demonstrated the highest binding tendencies are represented in Table 1. In the same way, the amino acid residues of the coronaviruses S protein that interacted with reference inhibitors and terpenoids with the highest binding affinity are shown in Table 2. The interacting residues of *human* ACE2 and TMPRSS2 with respective ligand groups were majorly through hydrophobic interactions and H-bond. Few H-bonding below 3.40 Å were observed with coronaviruses S protein (Table 1 and Fig. 3). The binding of MLN-4760 to ACE2 showed that it was docked into the N terminus and zinc-containing subdomain I of ACE2 (Fig. 4a). MLN-4760 exhibited several types of hydrophobic interactions (Pi-Sigma, Pi-Pi T-Shaped, Pi-Alkyl, and Alkyl) with TYR^510^ PHE^504^ MET^360^, LYS^363^, and CYS^344^, a salt and attractive charges to ARG^514^, ARG^518^, and ARG^278^ and hydrogen bond to TYR^515^, THR^371^, PRO^346^, and ARG^273^ (Fig. 4a). 24-methylene cycloartenol the best docked terpenoid was docked into the C terminus-containing subdomain II of ACE2 but interacted with different residue as with the case of N-acetyl-D-glucosamine (Fig. 4b). 24-methylene cycloartenol interacted via H-bond to TRP^163^, SER^170^, and TYR^497^. A Pi-Alkyl interaction was also observed with TYR^613^, PRO^492^, and VAL^491^. Isoiguesterin interacted via H-bond to ASP^350^, TYR^385^, and ASN^394^. A Pi-Alkyl and Alkyl interactions was observed with the ALA^99^, PHE^40^, PHE^390^, LEU^73^, and TRP^69^ residues respectively in a similar binding pattern with MLN-4760 (Fig. 4c). Camostat was docked into the S1-specificity pocket of TMPRSS2 (Fig. 5a). It interacted via conventional H-bond to five amino residues (ARG^41^, SER^195^, TRP^215^, ALA^190^, and ASP^189^) and via carbon hydrogen bond to GLN^192^ of TMPRSS2. The conventional H-bond was formed in the direction of the guanidine group in this order: first ester bond, second ester bond, while the last three residues interacted with amidino nitrogen of guanidine group, respectively. The phenyl ring was responsible for the carbon-hydrogen bond with GLN^192^ (Fig. 5a). T3 and T4 were docked into S1-specificity pocket of TMPRSS2 in a similar binding pattern as in the case of camostat (Fig. 5b, c). The only difference observed between the binding pattern of T3 and T4 was an additional H-bond between T3 with ARG^41^ (Fig. 5b). Nelfinavir mesylates an inhibitor of SARS-CoV and MERS-CoV S protein interacted in its best docked conformation to the S protein of SARS-CoV-2 in a different manner. Nelfinavir mesylates was docked into the S2 subunit of SARS-CoV S protein (Fig. 7a). The same inhibitor was docked into to the N-terminal domain (NTD) region of the S1 subunit of SARS-CoV-2 and MERS-CoV S protein (Figs. 6a and 8a). 3-benzoylhosloppone with the highest binding affinity for SARS-CoV-2 S protein interacted via H-bond to THR^547^; Alkyl interaction to PHE^541^ and Pi-Alkyl interaction to PRO^589^ and LEU^546^. The region of interaction was between the CTD and SD1 region of S1 subunit of SARS-CoV-2 S protein. Cucurbitacin B was docked to the S2 subunit of SARS-CoV-2 S protein but interacted with different amino acid residue. The interaction of cucurbitacin B to the protein was via H-bond to ARG^1091^, ASN^914^, THR^912^, and GLN^1113^; Pi-Sigma bond to PHE^1121^ and Alkyl interaction to ILE^1114^ and GLY^1124^ (Fig. 6c). The same pattern of interaction was observed in both 7-Deacetoxy-7-oxogedunin and 3-friedelanone to the S2 subunit of SARS-CoV S protein. Both terpenoids interacted via a H-bond to ARG^982^ and GLY^726^ of the S2 subunit. While 7-deacetoxy-7-oxogedunin interacted with the upstream helix and central helix, 3-friedelanone interacted with the connecting region of the S2 subunit. A hydrophobic interaction via Pi-Alkyl and alkyl bonds was observed with the remaining amino acid residue (Table 2; Fig. 7b, c). 7-Deacetoxy-7-oxogedunin interacted via H-bond to the SER^51^ residue of N-terminal domain of the S1 subunit of MERS-CoV S protein. A Pi-Pi T-shaped interaction was formed between 7-deacetoxy-7-oxogedunin and PHE^354^; HIS^670^ of MERS-CoV S protein. Other hydrophobic interactions via Pi-Alkyl and Pi-Sigma bonds were observed to with the remaining amino acid residues (Table 4; Fig. 8a, b). 3-benzoylhosloppone interacted via: Pi-Sigma interaction to (PHE^341^) of NTD; Pi-Pi Stacking to (MET^698^) of SD2; Pi-Alkyl interaction to (LYS^689^) of SD2; and an Alkyl interaction to (LEU^344^ and ILE^337^) of NTD with the S1 subunit of MERS-CoV S protein (Fig. 8c). In summary, the binding of ligands to various proteins revealed eight terpenoid with remarkable binding affinities. Those with very good interactions with ACE2 and TMPRSS2 are 24-methylene cycloartenol; isoiguesterin; 11-hydroxy-2-(3,4-dihydroxybenzoyloxy) abieta-5,7,9(11),13-tetraene-12-one; and 11-hydroxy-2-(4-hydroxybenzoyloxy)-abieta-5,7,9(11),13-tetraene-12-one. Similarly, 3-benzoylhosloppone, and cucurbitacin B interacted well with SARS-CoV-2 spike protein, while 7-deacetoxy-7-oxogedunin and 3-friedelanone interacted well with SARS-CoV and MERS-CoV spike protein.

**Table 1:** Interacting amino acid residue of *human* ACE2 and TMPRSS2 with the top binding terpenoids from African phytochemicals

Bioactive compound	Human protein targets	Interacted residues	Protein atom involved in H-bonding (bond distance)
S1 (MLN-4760)	ACE2	ARG^514^ ARG^518^ ARG^278^ TYR^510^ PHE^504^ MET^360^ LYS^363^ CYS^344^	TYR^515^(3.44) THR^371^ (3.03) PRO^346^ (3.08) ARG^273^ (2.93)
24-methylene cycloartenol **(T1)**		TRP^163^ SER^170^ TYR^497^ TYR^613^ PRO^492^ VAL^491^ SER^167^	TRP^163^ (3.22) SER^170^ (2.81) TYR^497^ (3.27)
Isoiguesterin (**T2)**		ASP^350^ TYR^385^ASN^394^ ALA^99^ PHE^40^ PHE^390^ LEU^73^ TRP^69^	ASP^350^ (3.27) TYR^385^ (3.27) ASN^394^ (3.27)
S2 (camostat)	TMPRSS2	ARG^41^ SER^195^ ALA^190^ ASP^189^ TRP^215^ GLN^192^	ARG^41^ SER^195^ ALA^190^ ASP^189^ TRP^215^
11-hydroxy-2-(3,4-dihydroxybenzoyloxy)abieta-5,7,9(11),13-tetraene-12-one **(T3)**		ARG^41^ GLN^192^ SER^195^ ALA^190^ ASP^189^ CYS^191^ HIS^57^ CYS^191^	ARG^41^ (2.41)GLN^192^ (2.89)SER^195^ (2.89)ALA^190^ (2.65)ASP^189^(2.39)
11-hydroxy-2-(4-hydroxybenzoyloxy)-abieta-5,7,9(11),13-tetraene-12-one **(T4)**		GLN^192^ ASP^189^ ALA^190^ SER^195^ HIS^57^ SER^214^ TRP^192^ CYS^219^	GLN^192^ (2.32) ASP^189^ (2.62) ALA^190^ (2.27) SER^195^ (2.32)

**Table 2:** Interacting amino acid residue of Spike protein of coronaviruses with the top binding terpenoids from selected African phytochemicals

Bioactive compound	Coronavirus spike proteins	Interacted residues	Protein atom involved in H-Bonding (bond distance)
(S3) Nelfinavir mesylates	SARS-Cov-2	THR^886^ ASP^867^ PRO^869^ PRO^862^ VAL^860^ SER^730^ HIS^1058^	THR^886^ **(3.48)** ASP^867^ **(2.13)** SER^730^ **(2.57)** HIS^1058^ **(2.03)**
3-benzoylhosloppone (**T5)**		THR^547^ PHE^541^ LEU^546^ PRO^589^	THR^547^**(3.03)**
Cucurbitacin B (**T6)**		ARG^1091^ ASN^914^ THR^912^ GLN^1113^ PHE^1121^ ILE^1114^ GLY^1124^	ARG^1091^ **(2.93)** ASN^914^**(3.32)** THR^912^ **(2.95)** GLN^1113^ **(2.89)**
(S3) Nelfinavir mesylates	SARS-CoV	SER^556^ THR^535^ THR^559^ PHE^558^ PRO^575^ PHE^527^	SER^556^ **(2.14)** THR^535^ **(2.38, 2.59)** THR^559^ **(3.30)**
7-deacetoxy-7-oxogedunin (**T7**)		ARG^982^ GLY^726^ VAL^958^ PHE^837^	ARG^982^ **(2.73, 2.16)** GLY^726^ **(2.52)**
3-Friedelanone (**T8**)		ARG^982^ GLY^726^ VAL^958^ PHE^837^ VAL^945^ LYS^836^ LEU^948^ ASN^838^	ARG^982^ (3.23) GLY^726^ (3.03) ASN^838^ (3.12)
(S3) Nelfinavir mesylates	MERS-CoV	SER^51^ ARG^335^ HIS^348^ HIS^670^ LEU^344^ ILE^337^ PHE^354^ LYS^668^	SER^51^ (2.90) ARG^335^ (2.89)
7-Deacetoxy-7-oxogedunin (**T7**)		SER^51^ HIS^348^ HIS^670^ ILE^337^ PHE^354^ LEU^344^ ARG^335^	SER^51^ (2.74)
3-Benzoylhosloppone		LYS^689^ PHE^341^ MET^698^ VAL^958^ LEU^344^ ILE^337^

#### Energy profile of best docked terpenoids to respective proteins

The overall energy profiles of terpenoid-protein complexes in the selected clusters with the best docked poses are shown in Figures S1– (supplementary data). Figure S1a–a (supplementary data) shows the breakdown of the binding energy of the selected cluster into different contributions. Gauss 1 (blue) and 2 (leaf green) bars represent the non-bonding interactions, red bar: repulsion, light blue bar: hydrophobic, purple bar: hydrogen bonds, light green bar: rotational forces, while the black bar represents total binding affinity which is a representative contribution of all bonding and non-bonding interactions between the terpenoids and the protein residues. The contributions of the various type of interaction as presented in graph (Figures S1a–a: supplementary data) shows that of the total binding energy of − 9.7 Kcal/mol exhibited by the binding of 24-methylene cycloartenol to the ACE2, − 2.1 and 1.8 Kcal/mol of hydrophobic and H-bond energies respectively was contributed, while the rest were contributed by non-bonding interaction mainly van der Waals, repulsive, and rotational forces. A H-bond, hydrophobic interaction, and repulsive energy of − 2.8 − 0.8, and + 2.3 Kcal/mol respectively was contributed to the total binding energy of − 10.0 Kcal/mol between T3 and TMPRSS2. Hydrophobic interaction affinities of − 2.1, − 0.6, and − 1.5 Kcal/mol, an H-bond energies of 0.3, − 0.6, and − 0.3 Kcal/mol were contributed to the total binding energy of the spike protein of SARS-CoV-2, SARS-CoV, and MERS-CoV with respective terpenoids. The rest of the energy was contributed by non-binding interactions.

Figures S1b–b (supplementary data) shows the overall energy profile of the ligand-receptor complex of the selected cluster, showing the individual energetic contributions for each atom in the ligand.

### Molecular dynamics simulation

**Fig. 9:**
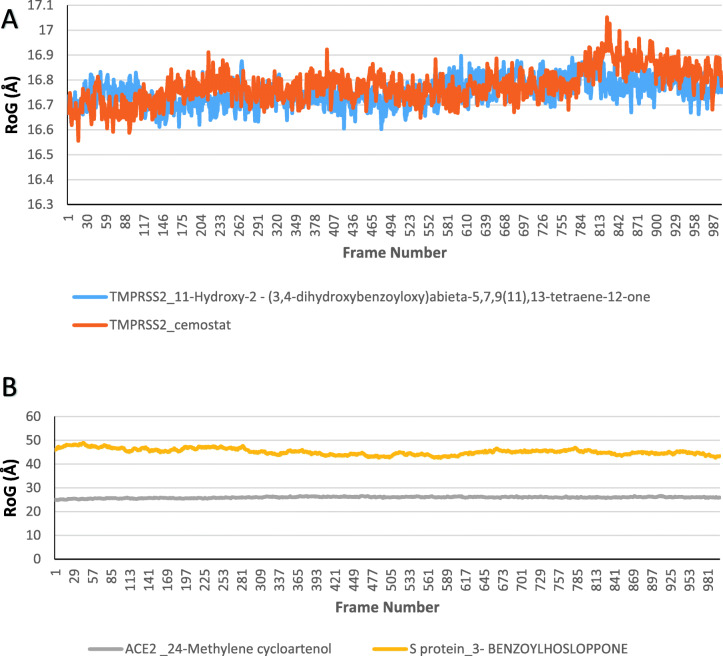
The radius of gyration plots for **a** TMPRSS2_camostat and TMPRSS2_11-hydroxy-2-(3,4-dihydroxybenzoyloxy)abieta-5,7,9(11),13-tetraene-12-one and **b** ACE2_24-methylene cycloartenol and S protein-3-benzoylhosloppone complexes

**Fig. 10:**
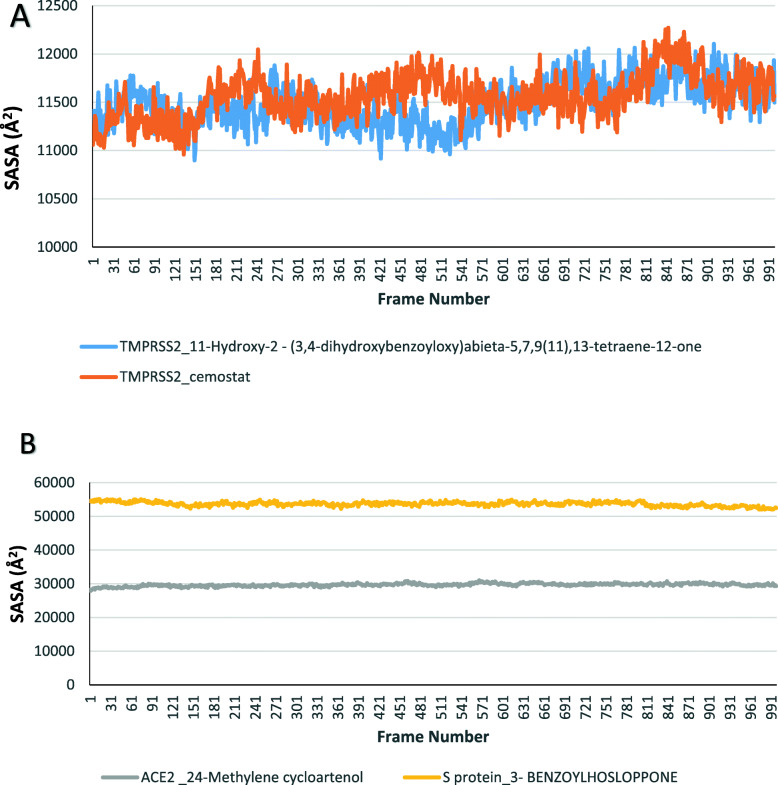
The surface accessible surface area (SASA) plots for **a** TMPRSS2_camostat and TMPRSS2_ 11-hydroxy-2-(3,4-dihydroxybenzoyloxy)abieta-5,7,9(11),13-tetraene-12-one and **b** ACE2_24-methylene cycloartenol, SARS-CoV-2 S protein-3-benzoylhosloppone complexes

**Fig. 11:**
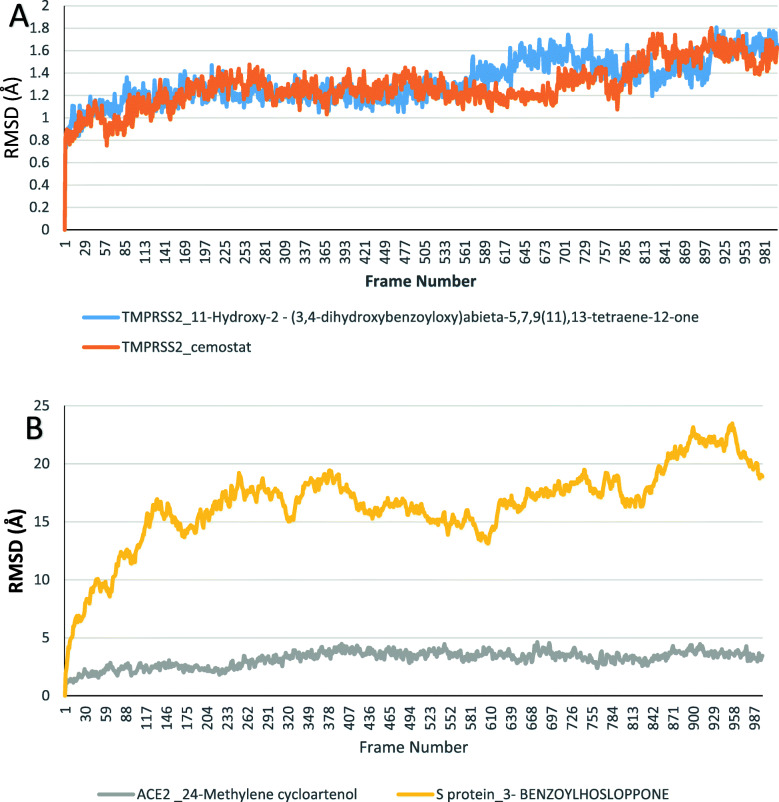
The root mean square deviation (RMSD) plots for **a** TMPRSS2_camostat and TMPRSS2_11-hydroxy-2-(3,4-dihydroxybenzoyloxy)abieta-5,7,9(11),13-tetraene-12-one and **b** ACE2_24-methylene cycloartenol and S protein-3-benzoylhosloppone complexes

**Fig. 12:**
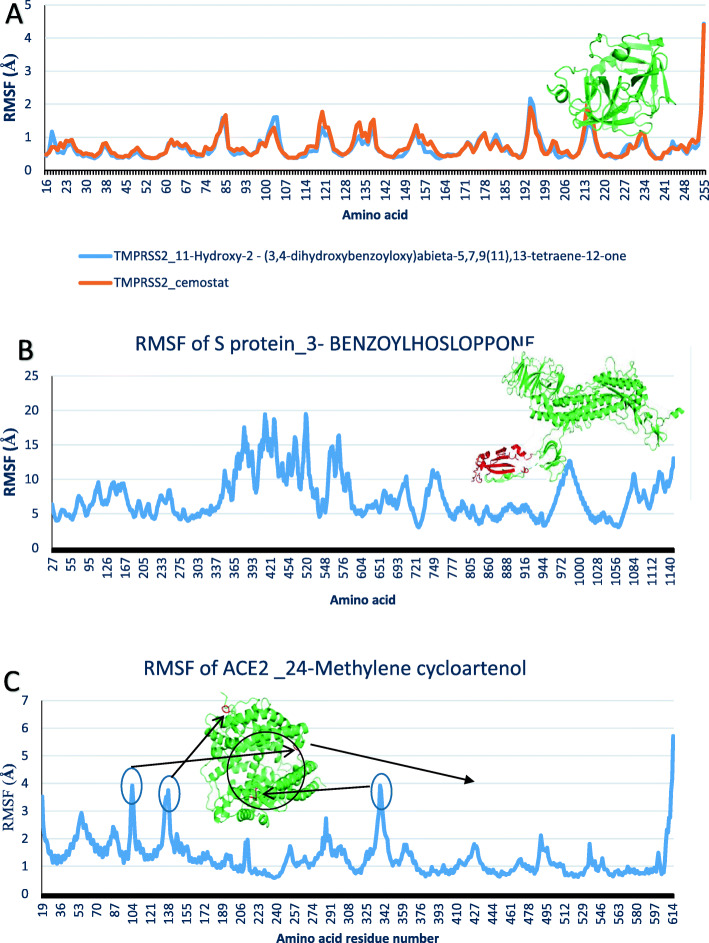
The root mean square fluctuation plots for **a** TMPRSS2_(11-hydroxy-2-(3,4-dihydroxybenzoyloxy)abieta-5,7,9(11),13-tetraene-12-one) and TMPRSS2_camostat. **b** SARS-CoV-2 S protein_(3-benzoylhosloppone). **c** ACE2_(24-methylene cycloartenol) respectively

Four compounds including camostat, T3, 24-methylene cycloartenol, and 3-benzoylhosloppone were analyzed for their interactions with transmembrane protease serine 2 **(**TMPRSS2), and Angiotensin-converting enzyme 2 (ACE2) and SARS-CoV-2 Spike glycoprotein (S protein). Molecular dynamics simulation was done on each of the target protein-terpenoids complexes and the trajectories were analyzed. The radius of gyration (RoG), root mean square deviation (RMSD), root mean square fluctuation (RMSF), and surface accessible surface area (SASA) results were calculated for each trajectory. The RoG values give indication on the folding/unfolding of the protein. There was no observed difference between the RoG of TMPRSS2_camostat and TMPRSS2_T3 complexes (Fig. 9a). The TMPRSS2_cemostat, TMPRSS2_T3, and ACE2_ 24-methylene cycloartenol complexes show a steady fluctuation around mean values of 16.77 Å, 16.75 Å, 25.95 Å, while the RoG values of the S protein_3-benzoylhoslopponecomplex are the most fluctuating. The RMSD values show the deviation of each frame from the initial configuration (Fig. 9a, b). The average RMSD values from the plots of the TMPRSS2_camostat (2.13 Å) and TMPRSS2_T3 (2.14 Å) system were very close, while the ACE2-24_methylene cycloartenol and S protein_3-benzoylhosloppone complexes are around 3.6 Å and 16.78 Å, respectively (Figs. 10 and 11). The SASA plots indicate the rate of conformational changes in the protein based on its solvent accessibility. TMPRSS2_cemostat, TMPRSS2_T3, ACE2_24-methylene cycloartenol, and S protein 3-benzoylhosloppone complexes have average values of 11563 Å^2^, 11498 Å^2^, 29667 Å^2^, and 53680 Å^2^ (Fig. 10). The RMSF plots give information on the fluctuation of individual amino acids. All the four complex systems have spikes at the end of RMSF plots that indicates the motion of the terminals. The mean RMSF values for TMPRSS2_camostat and TMPRSS2_T3 are 0.68 and 0.73 Å (Fig. 12a), while the ACE2_24-methylenecycloartenol and S protein_(3-benzoylhosloppone) complexes were fluctuating around 1.29 Å and 7.36 Å, respectively (Fig. 12b). The spikes in the middle and the start of the RMSF of ACE2_(24-methylene cycloartenol) complex between amino acid 265 and amino acid 443 and spikes in S protein_(3-benzoylhosloppone) complex corresponds to the loops in the two protein respectively (Fig. 12).

### Clustering analysis of the MD simulation trajectory of complexes

Table S3 (Supplementary data) show the number of clusters, interaction number, and types for TMPRSS2_T3, TMPRSS2_cemostat, S protein_3-benzoylhosloppone, and ACE2_24-methylene cycloartenol, respectively. Hydrophobic, H-bond, and salt-bridges interactions were observed from PLIP webserver. Figure S4 (supplementary data) shows the first and last cluster representatives for the protein-terpenoids complexes and the mode of interaction in the enlarged part of the image. Images were generated using PyMol software V 2.2.2.

### Molecular mechanics/generalized born surface area and decomposition analysis

**Fig. 13:**
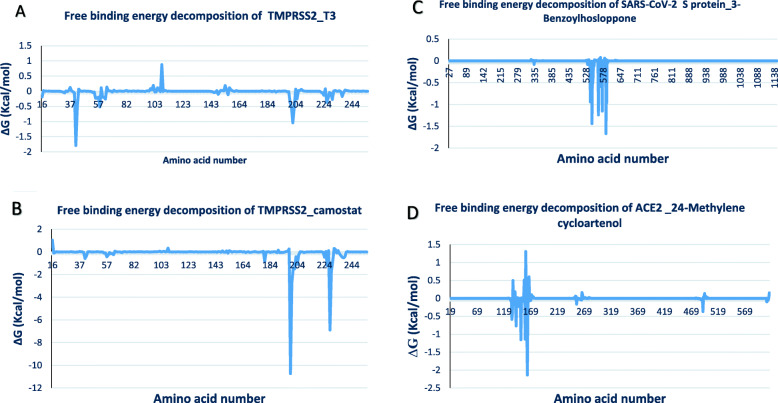
Molecular mechanics/generalized born surface area (MM/GBSA) plot of binding free energy contribution per residue of **a** TMPRSS2_11-hydroxy-2-(3,4-dihydroxybenzoyloxy)abieta-5,7,9(11),13-tetraene-12-one. **b** TMPRSS2_camostat. **c** SARS-CoV-2 S protein_3-benzoylhosloppone. **d** ACE2 _24-methylene cycloartenol

MM/GBSA free energy decomposition analysis was employed to decompose the total binding free energies (ΔGbind) into terpenoid-residue pairs, which would provide more detailed information regarding the contribution of each residue for ligand binding. It is obvious that the residue spectrograms of the TMPRSS2 systems were similar, though with different intensity of interactions. The high binding free energy of reference inhibitor (camostat) to TMPRSS2 was predominately through its interaction with APS^199^ and ASP^228^. Other H-bonds (ALA^190^, ASP^189^, and ALA^192^) contributed immensely to the free energy. The top docked terpenoid (T3) had stronger binding affinities to the residues ARG^41^ of TMPRSS2 than camostat. Both spectra show fluctuations around ARG41. The results of the energetic calculations that is presented in Table 3 show that the two TMPRSS2 systems had close values for ∆*E*_vdw,_ ∆*G*_ELE_, and ∆*G*_SA_. The high ∆*G*_total_ of camostat_ TMPRSS2 as compared to the T3_ TMPRSS2 may have been contributed by the ∆E_ele_ and ∆G_GB_. The decomposition plot for the ACE2 _24-methylene cycloartenol system and the SARS-CoV-2 S protein_3-benzoylhosloppone are in agreement with the results from the static docking analysis. The free binding energy of 24-methylene cycloartenol to ACE2 was majorly contributed by the H-bonds to SER^167^ and SER^170^ with the free energy contributions of which were greater than 1 kcal/mol. Other hydrogen bonds TRY^497^ and hydrophobic contacts to VAL^491^ were observed on the plot. The binding free energy of 3-benzoylhosloppone to SARS-Cov-2 S protein was majorly contributed by the H-bond and hydrophobic contact to THR^547^ other contributing residues includes LEU^546^, PHE^565^, VAL^576^, and ILE^587^ (Fig. 13).

**Table 3:** Binding free energies (∆*G* = Kcal/mol) and individual energy terms from MMGBSA analysis for target protein-terpenoids complexes

system	∆*E*_vdw_	∆*E*_ele_	∆*G*_GB_	∆*G*_SA_	T∆S	∆*G*_total_
S Protein_3-Benzoylhosloppone	− 49.66 ± 5.79	− 2.94 ± 3.6	16.9 ± 4.15	− 4.82 ± 0.85	− 15.43 ± 1.21	− 24.52 ± 5.06
Ace2_ 24-methylene cycloartenol	− 40.37 ± 5.60	− 5.03 ± 6.90	26.39 ± 7.20	− 4.37 ± 0.80	− 8.23 ± 1.11	− 15.39 ± 4.05
TMPRSS2_camostat	− 44.02 ± 5.41	− 224.83 ± 13.24	208.65 ± 11.05	− 5.28 ± 0.58	− 12.23 ± 2.02	− 53.70 ± 5.01
TMPRSS2_T3	− 42.53 ± 4.31	− 8.74 ± 8.62	28.45 ± 7.30	− 4.18 ± 0.43	− 11.21 ± 1.15	− 16.00 ± 4.08

*T3* = 11-Hydroxy-2-(3,4-dihydroxybenzoyloxy) abieta-5,7,9(11),13-tetraene-12-on

### Drug likeness and pharmacokinetic properties of selected terpenoids

The result generated from the Lipinski and ADMET filtering analyses are represented in Table 4 and Figure S5 (supplementary file). Four terpenoids T1, T3, T5, and T6 fulfilled the requirement for Lipinski analysis of the rule-of-five with corresponding favorable predicted ADMET parameters. The in silico predictive pharmacokinetic and ADMET properties from the filtering analyses suggested T1, T3, T5, and T6 with a high probability of absorption, subcellular distribution, and low toxicity. Though pharmacokinetic analysis indicated T1 (Table 4) to be less soluble while the ADME/tox analysis indicated high aqueous solubility, ability to pass the high human intestinal absorption, low acute oral toxicity with a good bioavailability score as exhibited by T3, T5, and T6 (Table 4).

**Table 4:** Physicochemical properties of the top binding terpenoids from African plants to ACE2, TMPRSS2, and S protein of SARS-Cov-2

a) Lipinski filter analysis				
Lipinski filters	**T1**	**T3**	**T5**	**T6**
Molecular weight (g/mol)	454.77	450.52	402.48	558.70
Num. heavy atoms	33	33	30	40
Num. rotatable bonds	5	4	4	6
Num. H-bond acceptors	1	6	4	8
Hydrogen bond donor	1	3	0	3
MLogP	7.30	2.96	3.79	1.76
Molar refractivity	144.50	126.11	116.15	150.94
Lipinski violation	1	0	0	1
(b) admet SAR				
Absorption (probability)				
Blood-brain barrier	BBB+ (0.96)	BBB+ (0.60)	BBB+ (0.61)	BBB+ (0.81)
Human intestinal absorption	HIA+ (0.99)	HIA+ (0.92)	HIA+ (0.92)	HIA+ (0.97)
Bioavailability score	0.55	0.55	0.55	0.55
Caco-2 permeability	Caco2+ (0.79)	Caco2+ (0.59)	Caco2+ (0.59)	Caco2+ (0.61)
P-glycoprotein substrate	Substrate (0.73)	Substrate (0.78)	Non-inhibitor (0.58)	Substrate (0.79)
P-glycoprotein inhibitor	Non-inhibitor (0.65)	Non-inhibitor (0.74)	Non-inhibitor (0.74)	Non-inhibitor (0.61)
Renal organic cation transporter	Inhibitor (0.75)	Inhibitor (0.90)	Non-inhibitor (0.90)	Non-inhibitor (0.87)
**Distribution** (probability)				
Subcellular localization	Lysosome (0.55)	Mitochondria (0.86)	Mitochondria (0.86)	Mitochondria (0.77)
**Metabolism**				
CYP450 substrate	Substrate (0.77) Non-inhibitor (0.78)	Substrate Non-inhibitor (0.83)	Non-substrate (0.65) inhibitor (0.80)	Inhibitor (0.79) Non-substrate (0.83)
**Toxicity**				
AMES toxicity	Non-AMES toxic (0.71)	AMES toxic (0.87)	Non-AMES toxic (0.87)	Non-AMES toxic (0.84)
Carcinogens	Non-carcinogens (0.92)	Non-carcinogens (0.90)	Non-carcinogens (0.90)	Non-carcinogens (0.92)
Acute oral toxicity	III (0.77)	III (0.59)	III (0.57)	I (0.78)
Rat acute toxicity LD_50_, mol/kg	3.2804	2.5370	2.5370	3.8742
Aqueous solubility (LogS)	− 4.76258	− 4.5550	− 4.7201	− 4.5035
**Pharmacokinetics**				
Lower GI absorption	Low	High	High	Low
Log *K*_p_ (skin permeation) cm/s	− 1.48	− 5.58	− 5.33	− 7.83

*T1* 4-methylene cycloartenol, *T3* 11-hydroxy-2-(3,4-dihydroxybenzoyloxy)abieta-5,7,9(11),13-tetraene-12-one, T5 3-benzoylhosloppone, *T6* cucurbitacin B

## Discussion

The prediction of drug–target interactions especially in new proteins is an essential stage in the drug discovery and development process [33]. Interference with several proteins that mediate viral attachment, membrane fusion, and cell entry of coronaviruses is an emerging therapeutic strategy for preventing COVID-19 infection [7, 20]. This principle was earlier demonstrated with HIV [13, 19] and SARS-CoV [2]. Earlier screening and prospecting of therapeutic phytocompounds have been reported for both SARS-CoV and MERS-CoV [42, 46, 50, 65]. Cell-based assays have shown the antiviral potentials of specific plant terpenoids against severe acute respiratory syndrome coronavirus (SARS- CoV) [65, 70]. This study was therefore undertaken to identify plant-derived terpenoids with inhibitory potentials against membrane-mediated SARS-CoV-2 entry proteins. Specifically, two triterpenes namely 24-methylene cycloartenol and isoiguesterin were reported to target ACE2 as well as the host-virus interface (S-protein-ACE2 receptor complex). These compounds interacted with adjacent residues in the conserved domain, apparently portraying its ability to bind and block interactions of hotspot 31 residues. The residues near lysine 31, and tyrosine 41, 82–84, and 353–357 in human ACE2 are important for the binding of S-protein of coronavirus [28]. The hotspots, 31 and 353, make salt bridge between Lys31 and Glu35, and the hotspot 353, comprising a salt bridge between Lys353 and Asp38, and are both buried in hydrophobic environment; therefore, interaction within this region is suggested to affect the binding of its substrate [69]. In a similar study in which five selected phytochemicals from Chinese and Indian herbs, though the individual compounds interacted differently with the active site of ACE2, they tend to distort the conformation that is necessary for its binding to the viral S protein [4]. The binding interactions of 24-methylene cycloartenol and isoiguesterin to the Site-2 binding site of ACE2 were similar to the pattern exhibited by some repurposed drugs such as delapril and lisinopril perindopril [24]**.** Abietane diterpenes, namely 11-hydroxy-2-(3,4-dihydroxybenzoyloxy) abieta-5,7,9(11), 13-tetraene-12-one (T3), and 11-hydroxy-2-(4-hydroxybenzoyloxy)-abieta-5,7,9(11), 13-tetraene-12-one (T4) showed the strongest interaction with TMPRSS2. In a similar binding pattern to camostat, these compounds were fitted into the S1-specificity pocket. They interacted with residue ALA^190^, ASP^189^, and GLN^192^ that are known to be part of the amino acid found at the basement of the pocket. ASP^189^ at the bottom of the pocket is known to determine the specificity of the S1 pocket for basic residues Arg and Lys at position P1 of the substrate [26]. The result showed that the hydroxybenzoyloxyl moiety of the terpenoids (T3 and T4) was responsible for at least 75% of the H-Bond with the protein. It was further observed that just as in the case of benzamidine (the native ligand) and camostat, the hydroxybenzoyloxyl moiety of the two terpenoids points with its hydroxyl group towards the carboxylate group of ASP^189^ forming strong H-bonds with ASP^189^ and other residue in the pocket. For camostat, the phenylquanidine moiety pointed into the hydrophobic pocket with the negatively charged ASP^189^ at its bottom. Unlike the H-bond formed between the amidino nitrogen of the phenylquanidine and benzamidine, in T3 and T4 the H-Bonds were formed mainly with the hydroxyl and carboxylate group. A striking similarity observed was that the ester bond that linked both the phenylquanidine moiety of camostat and the hydroxybenzoyloxyl moiety of T3 and T4 to the remaining structural unit of the compounds formed strong H-Bonds to the same residue SER^195^_._ The phenyl group of the hydroxybenzoyloxy moiety of T3 and T4 further interacted with hydrophobic interactions to CYS^119^ and CYS^219^ just as the peptide planes of the bonds between TRP^215^–GLY^216^ and CYS^191^–GLN^192^ sandwich the phenyl ring of benzamidine [16, 26]. The additional hydrophobic interaction by T3 and T4 may have been responsible for the exhibited higher binding affinities than camostat and benzamidine. Furthermore, while the hydroxybenzoyloxy moiety was directed towards the hydrophobic cleft created by ASP^189^, the abietane agylcon interacted with the imidazol ring of HIS^57^ of the S2 pocket that is found next to the S1 pocket and ARG^41^ (in the case of T4) which are outside the hydrophobic cleft. A similar interaction as the later was observed with camostat. The strong similarity in the binding pattern and even a far strong binding affinity than camostat and benzamidine indicates that T3, T4, and other abietane diterpenes especially those with hydroxybenzoyloxyl moiety attached to the abietane aglycon are potential inhibitors of TMPRSS2, thus preventing some coronaviruses from entering host [26]. Some natural compounds were found to interact with the protease furan of TMPRSS2, and these compounds exhibited different binding modes in the active site [52, 62]. It is known that, like SARS-CoV, SARS-CoV-2 S protein recognizes and binds to host-cell receptor angiotensin-converting enzyme 2 (ACE2) using a transmembrane protease serine 2 (TMPRSS2) which activates the S protein to facilitate viral fusion and entry into cells [68]. It is important to note that serine protease inhibitors like camostat mesylate, which blocks the activity of TMPRSS2 [77], has been approved in Japan for human use. Related compounds with antiviral activity potentiates as anti-SARS-CoV-2 agent [71]. Also, some abietane terpenoids have been identified to exhibit in vitro anti-SARS-CoV activity [65]. This corroborates the result of our study that shows that abietane diterpenes exhibits a wide spectrum and multiplicity of protein binding and may thereby specifically execute a complete blockage of viral entry. With regard to coronavirus S-proteins, 3-benzoylhosloppone and cucurbitacin B were the two terpenoids of utmost interest. While 3-benzoylhosloppone interacted with amino acid residue of the RBD and SD1 region of the S1 subunit, cucurbitacin B was docked into the S2 subunit of SARS-CoV-2 S protein. The former subunit is responsible for receptor recognition while the later mediates the fusion of viral membrane and the host cellular membrane [76]. Some phytochemicals known to interact with the RBD region and other binding site of the SARS-CoV-2 S protein have been reported to disrupt the binding of the S protein to the ACE2 protein [4, 45]. These terpenoids may prevent interaction of spike protein with its host cell receptor, thereby preventing entry of virus into host cell. 3-benzoylhosloppone has been reported for its antimalarial property while cucurbitacin B is an anticancer agent [1, 15].

Molecular dynamics (MD) simulations was performed after docking analysis to assess the physical transitions of atoms to effectively adopt the structure-to-function relevance of top docked terpenoids-target proteins and to further understand the dynamic behavior of the top docked terpenoids in the binding site of the various conformations of the target protein complexes in a dynamic environment [75]. The stability and structural/conformational fluctuations that occurred in the target proteins-terpenoids systems were monitored by clustering analysis of the MDS trajectory files. The RMSD is a plausible measure of protein stability. RMSD data shows how much each frame is deviated from the initial conformation of the reference structure as a function of time [11]. The comparison of the RMSD plots for the camostat_TMPRSS2 and T4_TMPRSS2 systems shows that the binding of T3 did not cause any structure deformation in TMPRSS2 as the binding of camostat. RMSF indicates the flexibility of different regions of a protein and the amino acid residue along the trajectory, which can be related to crystallographic B factors [11]. Though a lower amount of fluctuation occurred at with the interacting residues, it has been established that greater amounts of structural fluctuations usually occur in regions known to be involved in ligand binding and catalysis, notably the catalytic loop regions [14]. The RoG and SASA were assessed to evaluate the structural compactness and the accessibility of solvent to the proteins. A stably folded protein maintains a reasonably steady RoG over the simulation time. The stability of the complex is affected by loss of compactness through the introduction of weak intermolecular bonds [51].The RoG and SASA plots of all the systems did not show fluctuation that indicates deformation of the structural integrity of the proteins. The analyses of the thermodynamic parameters of the systems show that the top docked terpenoid complexed with respective proteins targets were stable and could be therefore subjected to experimental processes in further studies. At a quantitative level, simulation-based methods provide substantially more accurate estimates of ligand binding affinities (free energies) [43]. These results are calculated based on the total binding free energy of the complex. In these calculations, the binding free energy (∆*G*_bind_) measures the affinity of a ligand to its target protein. The free energy difference between the ligand-bound state (complex) and the corresponding unbound states of proteins and ligands are also employed in the calculations. Thus, the ∆*G*_bind_ calculations are important to gain in-depth knowledge about the binding modes of the hits in drug design [25]. The result from the MMPBSA calculation further corroborated the docking studies. The same amino acid residues were involved in the interactions with the top docked terpenoids in the static and dynamic states. From the Lipinski, pharmacokinetic, and ADMET filtering analyses, we identified four druggable and non-toxic, natural terpenoids that exhibited strong binding tendency to the various protein targets that mediates coronavirus-host cell entry. The result from the predicted filtering analyses of the four compounds showed parameters that suggest a favorable in silico ADMET and pharmacokinetic properties. The terpenoids expressed high probability of human intestinal absorption. They were also non-substrate to the permeability-glycoprotein (P-gp) [29], expressed capability to cross the blood brain barrier (BBB). SARS-CoV-2 has been reported to infect the brain, thus indicating its ability to cross the blood brain barrier (BBB) [73]. Therefore, compounds that can cross the BBB will be beneficial in the overal all viral clearance. The four terpenoids did not show indication of mutagenicity in silico, thereby they may not cause genetic mutations. The compounds did not display inhibitory potential for the various cytochrome P450, thus may not adversely affect phase I drug metabolism in the liver. These terpenoids are therefore considered as potential drug candidates.

## Conclusions

A virtual screening approach was successfully applied to identify plant-derived terpenoids as potential inhibitor of coronavirus cells entry proteins. Two pentacyclic terpenoids (4-methylene cycloartenol and isoiguesterin) interacted strongly with the binding sites residues that are known to interfere with the activity of ACE2. The abietane diterpene especially: 11-hydroxy-2-(3,4-dihydroxybenzoyloxy) abieta-5,7,9 (11), 13-tetraene-12-one (T3), and 11-hydroxy-2-(4-hydroxybenzoyloxy)-abieta-5,7,9(11), 13-tetraene-12-one (T4) exhibited a similar binding pattern to the S1-specificity pocket of TMPRSS2 as camostat (reference inhibitor). They also showed wide spectrum and multiplicity of entry protein binding. The terpenoids binding conformations in the complexes were stable in a simulated dynamic environment. The MM-GBSA binding free energy calculations corroborated the static docking analysis. Since the identified lead terpenoids showed drug-likeness and low toxicity as indicated by the in silico pharmacokinetically relevant molecular descriptors, they are postulated as potential inhibitors that can be considered for further in vitro and in vivo studies towards developing entry inhibitors against the ongoing coronavirus pandemic.

## Supplementary Information

**Additional file 1: Table S1**. Binding energies of bioactive terpenoids from African plants with higher affinity to human ACE2 and TMPRSS2, and SARS-Cov-2 S protein. **Table S2**. AutoDock scores (binding energies) of standard drugs and top 20 bioactive terpenoids with human Angiotensin-Converting Enzyme 2 (ACE2), Transmembrane Protease Serine 2 (TMPRSS2), and ACE2-Spike Receptor Binding Domain complex (ACE2-RBD). **Table S3**. AutoDock scores (binding energies) of standard drug and bioactive terpenoids from selected African phytochemicals to the spike protein of Coronaviruses. **Table S4**. Shows the number of clusters produced from TTClust, its representative frame for each of the protein-ligand complexes, and the interactions between the ligand and the protein from PLIP webserver for that frame. **Figure S1**. Energy profile of 24-methylene cycloartenol binding groups in human ACE2: (a) Energetic contribution to the Binding energy (d) Energetic contributions for each atom in the ligand. Number of poses in selected cluster: 68, best pose: 116 and binding site coordinate: 39.14, 35.33, and 12.71. **Figure S2**. Energy profile of T3 binding groups in human TMPRSS2: (a) Energetic contribution to the Binding energy (d) Energetic contributions for each atom in the ligand. Number of poses in selected cluster: 87, best pose: 40 and binding site coordinate: -2.96, 26.97, and 23.55. **Figure S3**. Energy profile of 3- benzoylhosloppone binding groups in SARS-Cov-2 S protein (a) Energetic contribution to the Binding (b) Energetic contributions for each atom in the ligand. Number of poses in selected cluster: 49, best pose: 571 and binding site coordinate: 214.85, 246.53, and 212.68. **Figure S4**. The representative structure for each cluster in cartoon representation, ligands in sticks representation and the types of interactions. Gray-dotted line: hydrophobic interactions, blue lines: H-bond interactions, yellow-dotted lines: salt-bridges interactions, and green-dotted lines: pi-stacking interactions. Single-letter amino acids are in red color. **Figure S5**. Summary of phamacokinetic properties of top binding terpenoids from African plants (a) T1: 24-methylene cycloartenol; (b) T3:11-Hydroxy-2 - (3,4-dihydroxybenzoyloxy) abieta -5,7,9(11),13-tetraene-12-one: (c) T5: 3- Benzoylhosloppone and (d) T6: Cucurbitacin B to the ACE2, TMPRSS2 and S protein of SARS-Cov-2.

## Data Availability

The authors confirm that the data supporting the findings of this study are available within the article [and/or] its supplementary materials.

## Abbreviations

COVID-19: Coronavirus disease-19
SARS-CoV-2: Severe acute respiratory syndrome coronavirus 2
SARS-CoV: Severe acute respiratory syndrome coronavirus
MERS-CoV: Middle East respiratory syndrome coronavirus
TMPRSS2: Transmembrane protease serine 2
ACE2: Angiotensin-Converting Enzyme 2
ADMET: Absorption, distribution, metabolism, excretion, and toxicity
MM-GBSA: Mechanics–generalized born surface area
RMSD: Root mean square deviation
RMSF: Root mean square fluctuation
SASA: Surface accessible surface area
RoG: Radius of gyration
PBC: Periodic boundary conditions
