# Supplementary material for: SARS-CoV-2 host cell entry: an in silico investigation of potential inhibitory roles of terpenoids
Source: J Genet Eng Biotechnol. 2021 Aug 5;19:113. doi: 10.1186/s43141-021-00209-z (PMC8339396; doi:10.1186/s43141-021-00209-z)
Supplement: Supplementary file 1 — Additional file 1: Table S1. Binding energies of bioactive terpenoids from African plants with higher affinity to human ACE2 and TMPRSS2, and SARS-Cov-2 S protein. Table S2. AutoDock scores (binding energies) of standard drugs and top 20 bioactive terpenoids with human Angiotensin-Converting Enzyme 2 (ACE2), Transmembrane Protease Serine 2 (TMPRSS2), and ACE2-Spike Receptor Binding Domain complex (ACE2-RBD). Table S3. AutoDock scores (binding energies) of standard drug and bioactive terpenoids from selected African phytochemicals to the spike protein of Coronaviruses. Table S4. Shows the number of clusters produced from TTClust, its representative frame for each of the protein-ligand complexes, and the interactions between the ligand and the protein from PLIP webserver for that frame. Figure S1. Energy profile of 24-methylene cycloartenol binding groups in human ACE2: (a) Energetic contribution to the Binding energy (d) Energetic contributions for each atom in the ligand. Number of poses in selected cluster: 68, best pose: 116 and binding site coordinate: 39.14, 35.33, and 12.71. Figure S2. Energy profile of T3 binding groups in human TMPRSS2: (a) Energetic contribution to the Binding energy (d) Energetic contributions for each atom in the ligand. Number of poses in selected cluster: 87, best pose: 40 and binding site coordinate: -2.96, 26.97, and 23.55. Figure S3. Energy profile of 3- benzoylhosloppone binding groups in SARS-Cov-2 S protein (a) Energetic contribution to the Binding (b) Energetic contributions for each atom in the ligand. Number of poses in selected cluster: 49, best pose: 571 and binding site coordinate: 214.85, 246.53, and 212.68. Figure S4. The representative structure for each cluster in cartoon representation, ligands in sticks representation and the types of interactions. Gray-dotted line: hydrophobic interactions, blue lines: H-bond interactions, yellow-dotted lines: salt-bridges interactions, and green-dotted lines: pi-stacking interactions. [file 43141_2021_209_MOESM1_ESM.docx]

**SARS-CoV-2 host cell entry: an *in silico* investigation of potential inhibitory roles of terpenoids**

Supplementary Data

**Table S1: Binding energies of bioactive terpenoids from African plants with higher affinity to human ACE2 and TMPRSS2, and SARS-Cov-2 S protein**

| **S/No** | **Bioactive Compounds** | **Class of compound** | **Plant species (Family)** | **ACE2** | **TMPRSS2** | SARS-Cov-2  S protein |
| --- | --- | --- | --- | --- | --- | --- |
|  |  |  |  | Binding affinity (Kcal/mol) | | |
| 1 | (13*S*)-Ent-7 -Hydroxy-3-cleroden-15-oic acid (**64**) | Clerodane and labdane diterpenoids | Nuxia sphaerocephala (Loganiaceae) | -7.2 | -6.2 | -6.9 |
| 2 | Ent-7 Hydroxy-2-oxo-3-cleroden-15-oic acid (**65**) | Clerodane and labdane diterpenoids | Nuxia sphaerocephala (Loganiaceae) | -7.3 | -6.8 | -7.5 |
| 3 | Ent-2,7-Dioxo-3-cleroden-15-oic acid (**66**) | Clerodane and labdane diterpenoids | Nuxia sphaerocephala (Loganiaceae) | -7.2 | -6.7 | -7.5 |
| 4 | Ent-18-*E*-Caffeoyloxy-7 -hydroxy-3-cleroden-15-oic acid (**67**) | Clerodane and labdane diterpenoids | Nuxia sphaerocephala (Loganiaceae) | -8.3 | -8.5 | -7.2 |
| 5 | (13*S*)-Ent-18-*E*-Coumaroyloxy-8(17)-labden-15-oic acid (**68**) | Clerodane and labdane diterpenoids | Nuxia sphaerocephala (Loganiaceae) | -8.1 | -8.0 | -7.8 |
| 6 | Ent-18-*E*-Caffeoyloxy-8(17)-labden-15-oic acid (**69**) | Clerodane and labdane diterpenoids | Nuxia sphaerocephala (Loganiaceae) | -8.3 | -8.3 | -8.1 |
| 7 | Ent-15-*E*-Caffeoyloxy-8(17)-labden-18-oic acid (**70**) | Clerodane and labdane diterpenoids | Nuxia sphaerocephala (Loganiaceae) | -7.3 | -7.0 | -8.6 |
| 8 | Aframodial (**71**) | Clerodane and labdane diterpenoids | Aframomum latifolium | -6.6 | -6.1 | -6.8 |
| 9 | (*E)*-8(17), 12-labddiene-15,16-dial (**72**) | Clerodane and labdane diterpenoids | Aframomum latifolium | -6.6 | -5.7 | -7.2 |
| 10 | (*E*)-15,15-diethoxylabda-8(17),12-dien-16-al (**73**) | Clerodane and labdane diterpenoids | Aframomum latifolium | -6.6 | -6.0 | -6.3 |
| 11 | Coronarin B (**74** | Clerodane and labdane diterpenoids | Aframomum latifolium | -7.4 | -6.8 | -7.7 |
| 12 | 16-Oxolabda-8(17),12(*E*)-dien- 15-oic acid (**75**): | Clerodane and labdane diterpenoids | Turreanthus africanus (Meliaceae) | -6.7 | -6.3 | -6.3 |
| 13 | methyl-14,15-epoxylabda-8(17), 12(*E*)-Diene-16-oate (**76** | Clerodane and labdane diterpenoids | Turreanthus africanus (Meliaceae) | -6.2 | -6.0 | -7.0 |
| 14 | Zambesiacolactone A (**79**) | Clerodane and labdane diterpenoids | Turreanthus africanus (Meliaceae) | -6.8 | --7.1 | -7.1 |
| 15 | Aulacocarpin A (**81**): | Clerodane and labdane diterpenoids | Aframomum zambesiacum (Zingiberaceae | -6.9 | -6.4 | -6.7 |
| 16 | Zambesiacolactone B (**80**) | Clerodane and labdane diterpenoids | Aframomum zambesiacum (Zingiberaceae | .7.7 | -6.2 | -7.2 |
| 17 | 3-Deoxyaulacocarpin A (**78**) | Clerodane and labdane diterpenoids | Aframomum zambesiacum (Zingiberaceae | -7.8 | -5.8 | -7.4 |
| 18 | Aulacocarpin A (**81**): | Clerodane and labdane diterpenoids | Aframomum zambesiacum (Zingiberaceae | -6.5 | -6.2 | -6.6 |
| 19 | Coranarin B (**82** | Clerodane and labdane diterpenoids | Aframomum | -7.7 | -6.8 | -7.4 |
| 20 | Galanal A (83) | Clerodane and labdane diterpenoids | Aframomum | -7.1 | -6.6 | -6.9 |
| 21 | Galanal B (84) | Clerodane and labdane diterpenoids | Aframomum arundinaceum (Zingiberaceae) | -7.4 | -6.6 | -7.1 |
| 22 | Galanolactone (**85**) | Clerodane and labdane diterpenoids | Aframomum arundinaceum (Zingiberaceae) | -7.4 | -6.7 | -6.9 |
| 23 | (*E*)- 8 ,17-Epoxylabd-12-ene-5,16 dial (**86**) | Clerodane and labdane diterpenoids | Aframomum arundinaceum (Zingiberaceae) | -8.0 | -7.0 | -6.7 |
| 24 | (*E*) Labda-8,12-diene-15,16 dial (**87**) | Clerodane and labdane diterpenoids | Aframomum arundinaceum (Zingiberaceae) | -5.9 | -5.7 | -7.0 |
| 25 | Methyl uguenesonate (**88**) | Limonoids | Vepris uguenensis (Rutaceae) | -8.4 | -7.6 |  |
| 26 | Methylangolensate (**89**) | Limonoids | Khaya grandifoliola (Meliaceae) | -7.6 | -7.6 | -7.2 |
| 27 | Gedunin (**90**) | Limonoids | Khaya grandifoliola (Meliaceae) | -8.5 | -8.8 | -8.4 |
| 28 | 7-Deacetylkhivorin (**91**): | Limonoids | Khaya grandifoliola (Meliaceae) | -8.1 | 7.6 | -7.6 |
| 29 | 1-Deacetylkhivorin (**92**) | Limonoids | Khaya grandifoliola (Meliaceae) | -7.7 | -7.6 | -7.8 |
| 30 | 6-Acetylswietenolide (**93**) | Limonoids | Khaya grandifoliola (Meliaceae) | -8.1 | -7.4 | -7.7 |
| 31 | - Acetoxydihydronomilin (**94**) | Limonoids | Entandrophragma angolense (Meliaceae | -8.5 | -7.1 | -7.6 |
| 32 | 7 -Obacunyl acetate (**95**) | Limonoids | Entandrophragma angolense (Meliaceae | -8.5 | -8.2 | -8.0 |
| 33 | 7-Deacetoxy-7-oxogedunin (**96**) | Limonoids | Ekebergia capensis (Zingiberaceae) | -9.2 | -8.4 | -9.0 |
| 34 | Ekeberin C1 (**97**) | Limonoids | Ekebergia capensis (Zingiberaceae) | -9.0 | -8.1 | -8.9 |
| 35 | Ekeberin C2 (**98**) | Limonoids | Ekebergia capensis (Zingiberaceae) | -8.0 | -7.4 | -8.1 |
| 36 | Ekeberin C3 (**99**) | Limonoids | Ekebergia capensis (Zingiberaceae) | -7.6 | -7.3 | -7.7 |
| 37 | Isoiguesterin (**100**) | Bisnorterpenes | Bisnorterpenes | -9.5 | -8.4 | -8.2 |
| 38 | 20-*Epi*-isoiguesterinol (**101**) | Bisnorterpenes | Bisnorterpenes | -9.2 | -7.7 | -7.9 |
| 39 | Isoiguesterinol (**102** | Bisnorterpenes | Bisnorterpenes | -9.0 | -8.0 | -8.3 |
| 40 | 6-Oxoisoiguesterin (**103**) | Bisnorterpenes | Bisnorterpenes | -9.5 | -8.1 | -8.8 |
| 41 | Deoxycaesaldekarin C (109) | Cassane furanoditerpenes | Caesalpinia volkensii (Leguminosae) | -7.4 | -7.1 | -7.6 |
| 42 | Caesaldekarin C (110) | Cassane furanoditerpenes | Caesalpinia volkensii (Leguminosae) | -7.1 | -6.9 | -7.3 |
| 43 | 7 -Formyloxy-6,12-dihydroxy-abieta- 8,12-Diene-11,14-dione (**111**) | Abietane diterpenes | Plectranthus hadiensis (Lamiaceae) | -7.8 | -7.5 | -6.9 |
| 44 | 7 -Acetoxy-6,12-dihydroxy-abieta- 8,12-Diene-11,14-dione(**112**) | Abietane diterpenes | Plectranthus hadiensis (Lamiaceae) | -7.3 | -6.6 | -7.3 |
| 45 | 11-Hydroxy-2 -(4-hydroxybenzoyloxy)-abieta- 5,7,9(11),13-tetraene-12-one (**113**) | Abietane diterpenes | Plectranthus lucidus (Lamiaceae) | -9.2 | -9.2 | -8.8 |
| 46 | 11-Hydroxy-2 - (3,4-dihydroxybenzoyloxy)abieta -5,7,9(11),13-tetraene-12-one (**114**) | Abietane diterpenes | Plectranthus ecklonii (Lamiaceae) | -8.8 | -10.0 | -8.2 |
| 47 | 11-Hydroxy- 19-(methyl-buten-2-oyloxy)-abieta -5,7,9 (11),13-tetraene-12-one (**115**) | Abietane diterpenes | Plectranthus purpuratus (Lamiaceae) | -8.1 | -6.5 | -7.3 |
| 48 | 11-Hydroxy-19-(4-hydroxy-benzoyloxy)-abieta -5,7,9(11),13-tetraene-12-one (**116**) | Abietane diterpenes | Plectranthus purpuratus (Lamiaceae) | -8.2 | -7.7 | -9.0 |
| 49 | 11-Hydroxy-19-(3,4-dihydroxybenzoyloxy)- abieta-5,7,9(11),13-tetraene-12-one (**117**) | Abietane diterpenes | Plectranthus purpuratus (Lamiaceae) | -8.7 | -7.8 | 9.1 |
| 50 | Ferruginol (**118**) | Abietane diterpenes | Fuerstia africana (Lamiaceae) | -7.7 | -6.6 | -7.7 |
| 51 | 3- Benzoylhosloppone (**119** | Abietane diterpenes | Hoslundia opposita (Lamiaceae) | -8.4 | -8.0 | -9.2 |
| 52 | 13 -*Epi*-dioxiabiet-8(14)-en-18-ol (**120**) | Abietane diterpenes | Hyptis suaveolens (Lamiaceae) | -7.8 | -6.9 | -7.6 |
| 53 | Vernodalin (**121**) | Sesquiterpenes and sesquiterpene lactones | Vernonia amygdalina (Asteraceae) | -7.1 | -6.6 | -7.9 |
| 54 | Vernodalol (**122**) | Sesquiterpenes and sesquiterpene lactones | Vernonia amygdalina (Asteraceae) | -6.9 | -5.8 | -7.2 |
| 55 | Vernolide (**123**) | Sesquiterpenes and sesquiterpene lactones | Vernonia amygdalina (Asteraceae) | -8.1 | -6.7 | -8.4 |
| 56 | Hydroxyvernolide (**124**) | Sesquiterpenes and sesquiterpene lactones | Vernonia amygdalina (Asteraceae) | -7.3 | -6.8 | -7.5 |
| 57 | 16,17- Dihydrobrachycalyxolide (**125**) | Sesquiterpenes and sesquiterpene lactones | Vernonia brachycalyx (Asteraceae) | -8.8 | -6.8 | -7.3 |
| 58 | Ajugarin-1 (**126**) | Sesquiterpenes and sesquiterpene lactones | Ajuga remota (Lamiaceae) | -7.1 | -6.4 | -6.9 |
| 59 | 1(10)*E*,5*E*-germacradien-4 -ol (**127**) | Sesquiterpenes and sesquiterpene lactones | Reneilmia cincinnata (Zingiberaceae) | -6.3 | -5.5 | -6.3 |
| 60 | 5*E*,10(14)-germacradien-1 ,4 -diol (**128**) | Sesquiterpenes and sesquiterpene lactones | Reneilmia cincinnata (Zingiberaceae) | -6.6 | -5.6 | -6.8 |
| 61 | Oplodiol (**129**) | Sesquiterpenes and sesquiterpene lactones | Reneilmia cincinnata (Zingiberaceae) | -6.9 | -5.8 | -6.9 |
| 62 | 15-Acetoxy-8-[(2-methylbutyryloxy)]- 14-oxo-4,5-cis-acanthospermolide (**130**) | Sesquiterpenes and sesquiterpene lactones | Acanthospermum hispidum (Asteraceae) | -7.5 | -6.7 | -7.7 |
| 63 | 9-Acetoxy-15-hydroxy-8-(2- methylbutyryloxy)- 14-oxo-4,5-trans-acanthospermolide (**131**) | Sesquiterpenes and sesquiterpene lactones | Acanthospermum hispidum (Asteraceae) | -7.6 | -6.5 | -7.4 |
| 64 | Vernangulide A (**132**) | Sesquiterpenes and sesquiterpene lactones | Vernonia angulifolia (Asteraceae) | -6.9 | -6.0 | -7.5 |
| 65 | Vernangulide B (**133**) | Sesquiterpenes and sesquiterpene lactones | Vernonia angulifolia (Asteraceae) | -5.6 | -6.1 | -5.6 |
| 66 | Urospermal A-15-*O*-acetate (**136**) | Sesquiterpenes and sesquiterpene lactones | Dicoma tomentosa (Asteraceae | -6.8 | -6.9 | -7.0 |
| 67 | Artemisinin (**137**) | Sesquiterpenes and sesquiterpene lactones | Artemisia annua (Asteraceae) | -7.5 | -6.6 | -7.8 |
| 68 | Dehydrobrachylaenolide (**138**) | Sesquiterpenes and sesquiterpene lactones | Dicoma anomala subsp. gerrardii (Asteraceae) | -7.5 | -6.8 | -7.5 |
| 69 | Tagitinin C (**139**) | Sesquiterpenes and sesquiterpene lactones | Tithonia diversifolia (Asteraceae) | -7.3 | -6.4 | -7.9 |
| 70 | Okundoperoxide (**140**) | Sesquiterpenes and sesquiterpene lactones | Scleria striatinux (Cyperaceae) | -6.9 | -6.5 | -7.2 |
| 71 | 4(13),7-Coloratadiene-12,11-olide (**141)** | Coloratane sesquiterpenes | Warburgia ugandensis (Canellaceae) | -7.6 | -6.8 | -7.5 |
| 72 | 11 -Hydroxymuzigadiolide(**142**) | Coloratane sesquiterpenes | Warburgia ugandensis (Canellaceae) | -7.2 | -6.5 | -7.7 |
| 73 | Muzigadial (**143**) | Coloratane sesquiterpenes | Warburgia ugandensis (Canellaceae) | -6.4 | -5.7 | -6.9 |
| 74 | 6 ,9 -Dihydroxy-4(13),7- coloratadiene- 11,12-dial (**144**) | Coloratane sesquiterpenes | Warburgia ugandensis (Canellaceae) | -6.2 | -6.1 | -6.7 |
| 75 | Cinnamolide (**145**) | Coloratane sesquiterpenes | Warburgia ugandensis (Canellaceae) | -6.9 | -6.0 | -7.1 |
| 76 | Cinnamolide-3 -acetate (**146**) | Coloratane sesquiterpenes | Warburgia ugandensis (Canellaceae) | -7.2 | -6.3 | -7.5 |
| 77 | Mukaadial (**147**) | Coloratane sesquiterpenes | Warburgia ugandensis (Canellaceae) | -6.4 | -6.0 | -6.7 |
| 78 | Ugandensidial (**148**) | Coloratane sesquiterpenes | Warburgia ugandensis (Canellaceae) | -6.5 | -5.8 | -71 |
| 79 | Cryptobeilic acid A (**149**) | Beilshmiedic acid derivatives | Beilschmiedia cryptocaryoides (Lauraceae) | -7.2 | -6.1 | -7.6 |
| 80 | Cryptobeilic acid B (**150**) | Beilshmiedic acid derivatives | Beilschmiedia cryptocaryoides (Lauraceae) | -6.9 | -5.9 | -7.4 |
| 81 | Cryptobeilic acid C (**151**) | Beilshmiedic acid derivatives | Beilschmiedia cryptocaryoides (Lauraceae) | -8.9 | -7.8 | -9.2 |
| 82 | Cryptobeilic acid D(**152**) | Beilshmiedic acid derivatives | Beilschmiedia cryptocaryoides (Lauraceae) | -6.8 | -5.9 | -7.6 |
| 83 | Tsangibeilin B (**153**) | Beilshmiedic acid derivatives | Beilschmiedia cryptocaryoides (Lauraceae) | -8.3 | -7.6 | -8.3 |
| 84 | 3-Hydroxy-20(29)-lupen-28-ol (**154**) | Pentacyclic triterpenes | Schefflera umbellifera (Araliaceae) | -8.5 | -7.5 | -7.9 |
| 85 | Pristimerin (**155**) | Pentacyclic triterpenes | Maytenus senegalensis (Celastraceae) | -9.0 | -7.7 | -8.1 |
| 86 | 3-Oxolupenal (3-oxolup-20(29)-en-30-al) (**156**) | Pentacyclic triterpenes | Nuxia sphaerocephala (Loganiaceae) | -8.7 | -8.3 | -8.2 |
| 87 | 3 -Hydroxylupenal (3 -hydroxylup-20(29)-en-30-al) (**157**) | Pentacyclic triterpenes | Nuxia sphaerocephala (Loganiaceae) | -8.1 | -8.3 | -8.0 |
| 88 | 3-Oxolupenol (30-hydroxylup-20(29)-en-3-one) (**158**) | Pentacyclic triterpenes | Nuxia sphaerocephala (Loganiaceae) | -8.6 | -8.3 | -8.0 |
| 89 | 3 -Acetyloleanolic acid (**159**) | Pentacyclic triterpenes | Nuxia sphaerocephala (Loganiaceae) | -8.4 | -8.1 | -8.9 |
| 90 | Oleanolic acid (**160**) | Pentacyclic triterpenes | Nuxia sphaerocephala (Loganiaceae) | -9.7 | -8.3 | -8.3 |
| 91 | Lupeol (**161** | Pentacyclic triterpenes | Hymenocardia acida (Phyllanthaceae) | -8.2 | -7.7 | -8.1 |
| 92 |  | Pentacyclic triterpenes | Hymenocardia acida (Phyllanthaceae) |  |  |  |
| 93 | 22-Hydroxyhopan-3-one (**163**) | Pentacyclic triterpenes | Cassia siamea (Fabaceae) | -8.8 | -8.1 | -8.3 |
| 94 | 24-Methylene cycloartenol (**164**) | Pentacyclic triterpenes | Entandrophragma angolense (Meliaceae) | -9.7 | -8.4 | -8.6 |
| 95 | Betulinic acid (**165** | Pentacyclic triterpenes | Entandrophragma angolense (Meliaceae) | -8.5 | -7.5 | -7.3 |
| 96 | 3-Friedelanone (**166**) | Pentacyclic triterpenes | Hypericum lanceolatum (Hypericaceae) | -8.9 | -8.2 | -9.4 |
| 97 | 3-*O*-betulinic acid *p*-coumarate (**167**) | Pentacyclic triterpenes | Baillonella toxisperma (Sapotaceae) | -8.6 | -8.3 | -8.1 |
| 98 | 2 ,3 ,19 -Trihydroxy-urs-12-20-en-28-oic acid (**168**) | Pentacyclic triterpenes | Kigelia africana (Bignoniaceae) | -8.8 | -8.1 | -8.6 |
| 99 | Cucurbitacin B (**169**) | Pentacyclic triterpenes | Cogniauxia podolaena (Cucurbitaceae) | -8.4 | -7.6 | -9.3 |
| 100 | Cucurbitacin D (**170**) | Pentacyclic triterpenes | Cogniauxia podolaena (Cucurbitaceae) | -8.4 | -7.9 | -8.6 |
| 101 | 20-*Epi*bryonolic acid (**171**) | Pentacyclic triterpenes | Cogniauxia podolaena (Cucurbitaceae) | -8.9 | -8.7 | -9.1 |
| 102 | alpha_Pinene | Monoterpenes |  | -4.9 | -4.6 | -7.6 |
| 103 | alpha_Terpineol |  |  | -5.3 | -5.1 | -7.9 |
| 104 | Betulinaldehyde |  |  | -8.8 | -7.5 | -8.7 |
| 105 | Limonene |  |  | -6.3 | -5.0 | -4.6 |
| 106 | Tashironin |  |  | -8.3 | -6.8 | -7.6 |

### Table S2: AutoDock scores (binding energies) of standard drugs and top 20 bioactive terpenoids with human Angiotensin-Converting Enzyme 2 (ACE2), Transmembrane Protease Serine 2 (TMPRSS2), and ACE2-Spike Receptor Binding Domain complex (ACE2-RBD)

| **S/No** | **Bioactive Compounds** | **Class of compound** | **Plant species (Family)** | **Binding Affinity (Kcal/mol)** | | |
| --- | --- | --- | --- | --- | --- | --- |
|  |  |  |  | **ACE2** | **TMPRSS2** | **ACE2-RBD** |
| S1 | MLN-4760 |  |  | -7.0 |  |  |
| S2 | N-Acetyl-d-glucosamine |  |  | -5.6 |  |  |
| S3 | Camostat |  |  |  | **-7.6** |  |
| 1 | 24-Methylene cycloartenol | Pentacyclic triterpenes | Entandrophragma angolense (Meliaceae) | **-9.7** | -8.4 | -8.9 |
| 2 | Isoiguesterin | Bisnorterpenes | Bisnorterpenes | **-9.5** | -8.4 | -9.2 |
| 3 | 6-Oxoisoiguesterin | Bisnorterpenes | Bisnorterpenes | **-9.5** | -8.1 | **-9.5** |
| 4 | 11-Hydroxy-2 -(4 hydroxybenzoyloxy)-abieta- 5,7,9(11),13-tetraene-12-one | Abietane diterpenes | Plectranthus lucidus (Lamiaceae) | -9.2 | **-9.2** | -8.0 |
| 5 | 7-Deacetoxy-7-oxogedunin | Limonoids | Ekebergia capensis (Zingiberaceae) | -9.2 | -8.4 | -9.4 |
| 6 | 20-*Epi*-isoiguesterinol | Bisnorterpenes | Bisnorterpenes | -9.2 | -7.7 | -9.4 |
| 7 | Ekeberin C1 | Limonoids | Ekebergia capensis (Zingiberaceae) | -9.0 | -8.1 | -9.3 |
| 8 | Isoiguesterinol | Bisnorterpenes | Bisnorterpenes | -9.0 | -8.0 | -8.9 |
| 9 | Pristimerin | Pentacyclic triterpenes | Maytenus senegalensis (Celastraceae) | -9.0 | -7.7 | -9.0 |
| 10 | 20-*Epi*bryonolic acid | Pentacyclic triterpenes | Cogniauxia podolaena (Cucurbitaceae) | -8.9 | -8.7 | **-9.7** |
| 11 | 3-Friedelanone | Pentacyclic triterpenes | Hypericum lanceolatum (Hypericaceae) | -8.9 | -8.2 | -8.8 |
| 12 | Cryptobeilic acid C | Beilshmiedic acid derivatives | Beilschmiedia cryptocaryoides (Lauraceae) | -8.9 | -7.8 | -8.7 |
| 13 | 11-Hydroxy-2 - (3,4-dihydroxybenzoyloxy)abieta -5,7,9(11),13-tetraene-12-one | Abietane diterpenes | Plectranthus ecklonii (Lamiaceae) | -8.8 | **-10.0** | -8.9 |
| 14 | 2 ,3 ,19 -Trihydroxy-urs-12-20-en-28-oic acid | Pentacyclic triterpenes | Kigelia africana (Bignoniaceae) | -8.8 | -8.1 | -9.4 |
| 15 | 22-Hydroxyhopan-3-one | Pentacyclic triterpenes | Cassia siamea (Fabaceae) | -8.8 | -8.1 | -9.3 |
| 16 | 16,17- Dihydrobrachycalyxolide | Sesquiterpenes and sesquiterpene lactones | Vernonia brachycalyx (Asteraceae) | -8.8 | -6.8 | -7.0 |
| 17 | 3-Oxolupenal (3-oxolup-20(29)-en-30-al) | Pentacyclic triterpenes | Nuxia sphaerocephala (Loganiaceae) | -8.7 | -8.3 | -8.7 |
| 18 | 11-Hydroxy-19-(3,4-dihydroxybenzoyloxy)- abieta-5,7,9(11),13-tetraene-12-one | Abietane diterpenes | Plectranthus purpuratus (Lamiaceae) | -8.7 | -7.8 | -8.6 |
| 19 | 3-Oxolupenol (30-hydroxylup-20(29)-en-3-one) | Pentacyclic triterpenes | Nuxia sphaerocephala (Loganiaceae) | -8.6 | -8.3 | -8.5 |
| 20 | 3-*O*-betulinic acid *p*-coumarate | Pentacyclic triterpenes | Baillonella toxisperma (Sapotaceae) | -8.6 | -8.3 |  |

**Table S3:** AutoDock scores (binding energies) of standard drug and bioactive terpenoids from selected African phytochemicals to the spike protein of coronaviruses

| **S/No** | **Bioactive Compounds** | **Class of compound** | Plant species (Family) | Binding affinity (Kcal/mol) | | |
| --- | --- | --- | --- | --- | --- | --- |
|  |  |  |  | SARS-CoV-2 | SARS-CoV | MERS-CoV |
| S3 | Nelfinavir mesylates |  |  | **-7.7** | **-** **6.8** | **-8.8** |
| 1 | 3- Benzoylhosloppone | Abietane diterpenes | Hoslundia opposita (Lamiaceae) | **-9.4** | -8.9 | **-10.3** |
| 2 | Cucurbitacin B | Pentacyclic triterpenes | Cogniauxia podolaena (Cucurbitaceae) | **-9.3** | -7.8 | -9.4 |
| 3 | 3-Friedelanone | Pentacyclic triterpenes | Hypericum lanceolatum (Hypericaceae) | **-9.2** | **-9.4** | -9.1 |
| 4 | Cryptobeilic acid C | Beilshmiedic acid derivatives | Beilschmiedia cryptocaryoides (Lauraceae) | -9.2 | -7.8 | -9.9 |
| 5 | 20-*Epi*bryonolic acid | Pentacyclic triterpenes | Cogniauxia podolaena (Cucurbitaceae) | -9.1 | -8.9 | -10.1 |
| 6 | 11-Hydroxy-19-(3,4-dihydroxybenzoyloxy)- abieta-5,7,9(11),13-tetraene-12-one | Abietane diterpenes | Plectranthus purpuratus (Lamiaceae) | -9.1 | -8.3 | -8.9 |
| 7 | 7-Deacetoxy-7-oxogedunin | Limonoids | Ekebergia capensis (Zingiberaceae) | -9.0 | **-9.6** | -**10.6** |
| 8 | 11-Hydroxy-19-(4-hydroxy-benzoyloxy)-abieta -5,7,9(11),13-tetraene-12-one | Abietane diterpenes | Plectranthus purpuratus (Lamiaceae) | -9.0 | -7.7 | -9.1 |
| 9 | Ekeberin C1 | Limonoids | Ekebergia capensis (Zingiberaceae) | -8.9 | -9.4 | -9.1 |
| 10 | 3 -Acetyloleanolic acid | Pentacyclic triterpenes | Nuxia sphaerocephala (Loganiaceae) | -8.9 | -8.8 | -9.7 |
| 11 | 6-Oxoisoiguesterin | Bisnorterpenes | Bisnorterpenes | -8.8 | -9.1 | -9.1 |
| 12 | 11-Hydroxy-2 -(4-hydroxybenzoyloxy)-abieta- 5,7,9(11),13-tetraene-12-one | Abietane diterpenes | Plectranthus lucidus (Lamiaceae) | -8.8 | -8.1 | -8.3 |
| 13 | Ent-15-*E*-Caffeoyloxy-8(17)-labden-18-oic acid | Clerodane and labdane diterpenoids | Nuxia sphaerocephala (Loganiaceae) | -8.6 | -6.8 | -7.7 |
| 14 | 24-Methylene cycloartenol | Pentacyclic triterpenes | Entandrophragma angolense (Meliaceae) | -8.6 | -8.3 | -9.3 |
| 15 | 2 ,3 ,19 -Trihydroxy-urs-12-20-en-28-oic acid | Pentacyclic triterpenes | Kigelia africana (Bignoniaceae) | -8.6 | -9.2 | -9.3 |
| 16 | Cucurbitacin D | Pentacyclic triterpenes | Cogniauxia podolaena (Cucurbitaceae) | -8.6 | -8.4 | -8.9 |
| 17 | Gedunin | Limonoids | Khaya grandifoliola (Meliaceae) | -8.4 | -8.5 |  |
| 18 | Vernolide | Sesquiterpenes and sesquiterpene lactones | Vernonia amygdalina (Asteraceae) | -8.4 | -7.0 |  |
| 19 | Isoiguesterinol | Bisnorterpenes | Bisnorterpenes | -8.3 | -8.0 |  |
| 20 | Tsangibeilin B | Beilshmiedic acid derivatives | Beilschmiedia cryptocaryoides (Lauraceae) | -8.3 | -9.1 |  |
|  | S1 (MLN-4760) |  |  | -7.0 |  |  |
|  | S2 (N-Acetyl-d-glucosamine) |  |  | -5.6 |  |  |
|  | S3 Camostat |  |  |  | **-7.6** |  |


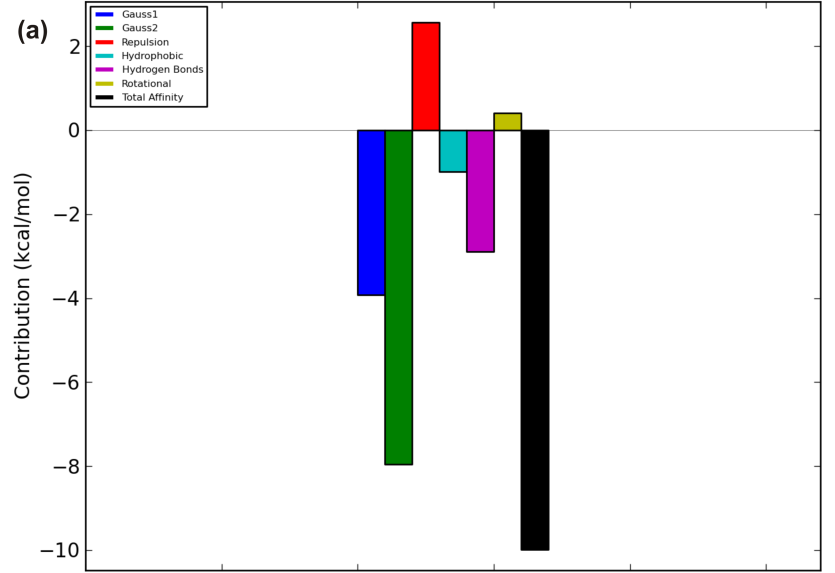


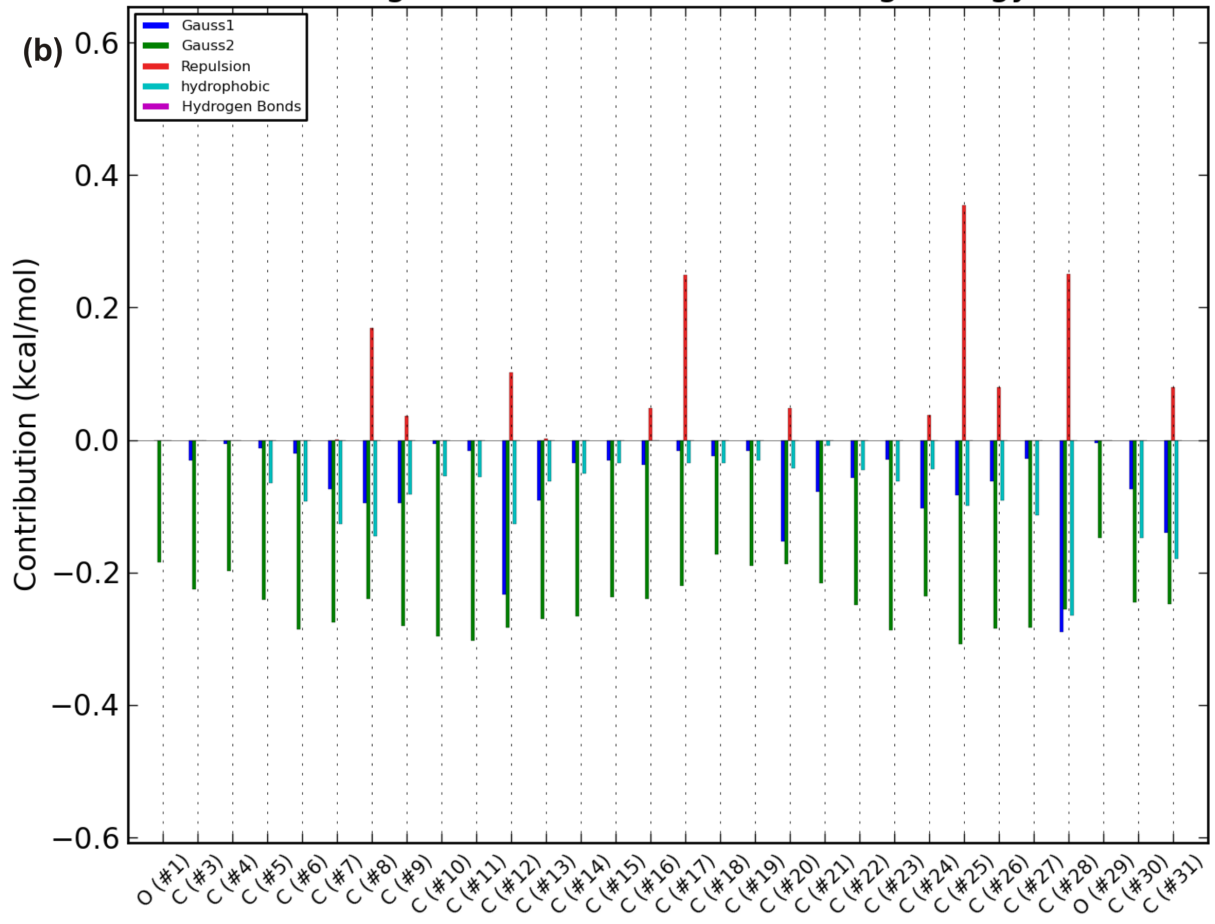


*Figure S1: Energy profile of 24-Methylene cycloartenol binding groups in human ACE2: (a) Energetic contribution to the Binding energy (d) Energetic contributions for each atom in the ligand. Number of poses in selected cluster:* *68, best pose: 116 and binding site coordinate:* *39.14, 35.33, and 12.71*

*Figure S2: Energy profile of T3 binding groups in human TMPRSS2: (a) Energetic contribution to the Binding energy (d) Energetic contributions for each atom in the ligand. Number of poses in selected cluster:* *87, best pose: 40 and binding site coordinate:* *-2.96, 26.97, and 23.55*

*Figure S3: Energy profile of 3- benzoylhosloppone binding groups in SARS-Cov-2 S protein (a) Energetic contribution to the Binding (b) Energetic contributions for each atom in the ligand. Number of poses in selected cluster:* *49, best pose: 571 and binding site coordinate:* *214.85, 246.53, and 212.68*

Table S4: Shows the number of clusters produced from TTClust, its representative frame for each of the protein-ligand complexes, and the interactions between the ligand and the protein from PLIP webserver for that frame.

| TMPRSS2_(11-Hydroxy-2 - (3,4-dihydroxybenzoyloxy)abieta-5,7,9(11),13-tetraene-12-one) complex | | | | |
| --- | --- | --- | --- | --- |
| CLUSTER NUMBER (REPRESENTATIVE FRAME) | **Hydrophobic** | **H-bond** | **Salt-bridges** | **Pi-cation** |
| Cluster 1 (frame 140) | W215 | A190 (2) | None | None |
| Cluster 2 (frame 853) | **R41** - T62 | S39 – H40 - **R41** | **R41** | **R41** |
| Cluster 3 (frame 977) | T61 | None | None | **R41** |
| ACE2 _(24-Methylene cycloartenol) complex | | | | |
| CLUSTER NUMBER (REPRESENTATIVE FRAME) | **Hydrophobic** |  |  |  |
| CLUSTER 1 (FRAME 172) | Y255 (2)- P612 |  |  |  |
| CLUSTER 2 (FRAME 721) | L142 (2) – I151 – L162 (2) |  |  |  |
| TMPRSS2_ camostat complex | | | | |
| cluster number (representative frame) | **Hydrophobic** | **H-bond** | **Salt-bridges** | **Pi-stacking** |
| Cluster 1 (frame 92) | None | **A190** (2) – Q192 – **D217** – E218 – A220 | **D189** | H57 |
| Cluster 2 (frame 618) | Q192 - V213 | R41 – **A190** – S195 – **D217** – E218 | H57 - **D189** | None |
| Cluster 3 (frame 284) | Q192 | **A190** (2) – S195 – **D217** (2) | **D189** | None |
| Cluster 4 (frame 728) | None | **A190** (2) – **D217** (2) | **D189** | None |
| Cluster 5 (frame 915) | Q192 | **A190** (2) – **D217** (2) – A220 | **D189** | None |
| S protein_(3- Benzoylhosloppone) complex | | | | |
| CLUSTER NUMBER (REPRESENTATIVE FRAME) | **Hydrophobic** | **H-bond** | **Pi-stacking** |  |
| CLUSTER 1 (FRAME 184) | L546 – **V576** – I587 (2) - P589 | None | None |  |
| CLUSTER 2 (FRAME 631) | F541 – **F543** – L546 – T549 - P589 | T573 | **F543** |  |
| CLUSTER 3 (FRAME 935) | F541 – **F543** – L546 – F565 – 573 - **V576** | None | None |  |

Amino acid residues were represented by single letter code. Bold amino acids are common in each protein-drug complex

*Figure S4: The representative structure for each cluster in cartoon representation, ligands in sticks representation and the types of interactions. Gray-dotted line: hydrophobic interactions, blue lines: H-bond interactions, yellow-dotted lines: salt-bridges interactions, and green-dotted lines: pi-stacking interactions. Single-letter amino acids are in red color.*

*The colour space is the suitable physiochememical space for oral bioavailability*

**LIPO** Lipophility: -0.7 < XLOGP3 < +5.0

**SIZE**: 150g/mol : < MW < 500g/mol

**POLAR** (Polarity): 20Å^2^ < TPSA < 130 Å ^2^

**INSOLU** (insolubility): 0 < Log S (ESOL) < 6

**INSATU** (insaturation): 0.25 < Fraction Csp3 <1

**FLEX** (Flexibity): 0 < Num. rotatable bonds < 9

### Figure S5: Summary of phamacokinetic properties of top binding terpenoids from African plants (a) T1: 24-Methylene cycloartenol; (b) T3:11-Hydroxy-2 - (3,4-dihydroxybenzoyloxy) abieta -5,7,9(11),13-tetraene-12-one: (c) T5: 3- Benzoylhosloppone and (d) T6: Cucurbitacin B to the ACE2, TMPRSS2 and S protein of SARS-Cov-2
